# Traditional knowledge and utilization of wild edible plants in Swat district, Pakistan: implications for nutrition and food security

**DOI:** 10.1186/s13002-026-00850-3

**Published:** 2026-02-04

**Authors:** Shujat Ali, Salahud Din, Sayed Afzal Shah, Wahid Hussain, Rainer W. Bussmann

**Affiliations:** 1 Independent Researcher, Swat KP, Pakistan; 2 University of Swat, Charbagh, Mingora Swat, KPK, Pakistan; 3 Department of Biological Sciences, National University of Medical Sciences (NUMS), Rawalpindi, Pakistan; 4 Department of Botany, Government Post Graduate College (GPGC), Parachinar, District Kurram, Khyber Pakhtunkhwa (KPK), Pakistan; 5 Department of Botany, State Museum of Natural History, Erbprinzenstrasse 13, 76133 arlsruhe, Germany; 6 Department of Ethnobotany, Institute of Botany and Bakuriani Alpine Botanical Garden, Ilia State University, 1 Botanical 18 Str., Tbilisi, Georgia

**Keywords:** Ethnobotany, Food security, Nutritional diversity, Sustainable livelihoods, Swat valley, Traditional knowledge, Wild edible plants

## Abstract

**Background:**

Wild edible plants are critical for local nutrition, cultural heritage, and livelihoods, yet their diversity and traditional uses are underexplored in Pakistan. Swat Valley, a biologically and culturally rich region, relies on wild plants for food security, particularly among economically vulnerable households.

**Methods:**

Field surveys were conducted from 2018 to 2022 across 20 villages in six tehsils of Swat District. A total of 160 informants (100 males, 60 females; aged 20–90 years) were interviewed using semi-structured questionnaires, in-depth interviews, and personal observations. The quantitative ethnobotanical indices, including (UV), (RFC), and (UR), were calculated to assess cultural importance and usage patterns.

**Results:**

A total of 175 wild edible plant species from 72 families were documented. Herbs dominated **(**62.3%**)**, followed by shrubs (20.6%**)**, trees **(**16%**)**, and climbers (1.1%**).** Young shoots **(**41.7%**)**, fruits **(**30.6%**)**, and leaves **(**13.9%**)** were most commonly used. Plants were used as vegetables **(**37%**)**, fruits **(**39%**)**, raw foods **(**14%**)**, teas/flavoring agents **(**5%**)**, sauces/chutneys (2%**)**, and oils **(**2%**).***Berberis lycium* showed the highest cultural importance (UV = 0.813, RFC = 0.75), followed by *Chenopodium album* (UV = 0.68, RFC = 0.63) and *Berberis vulgaris* (UV = 0.65, RFC = 0.63). Novel use analysis of 115 species revealed High Level (22%), Moderate Level (23%), and Low Level (55%) novelty. Twenty-seven species were marketed locally, generating income ranging from (0.09 to 1.74 $) per Kg. Conservation assessment highlighted Endangered species (*Abies pindrow*, *Mentha royleana*, *Zanthoxylum armatum*) and Vulnerable species (*Thymus linearis*, *Mentha longifolia*, *Morus alba*).

**Conclusion:**

Wild edible plants in Swat Valley provide essential nutrition, support local livelihoods, and sustain cultural traditions. Quantitative indices demonstrate the most culturally significant species and highlight gaps for conservation. Sustainable management, cultivation, and policy interventions are recommended to preserve these vital resources, ensuring biodiversity conservation and continued food security in mountainous regions.

## Introduction

 The term “wild food” refers to plant resources collected outside cultivated areas, including forests, grasslands, savannahs, and other unmanaged landscapes, where they contribute substantially to local diets [3]. Wild foods have long been integrated into the subsistence strategies of rural societies ranging from shifting cultivators and continuous croppers to hunter-gatherer communities, providing essential nutrients, medicinal value, and ecological resilience [2, 25]. Since ancient times, plants have served as primary sources of energy, nutrition, medicine, construction materials, and economic products, shaping human development across civilizations [3, 23, 51, 74]. Despite advances in agriculture, a large proportion of the global population continues to experience nutrient deficiencies, and wild edible plants (WEPs) are frequently used to supplement dietary gaps, especially in marginalized communities [4, 21, 23, 36, 37, 40, 67, 72, 74]. Although nearly half a million plant species exist worldwide, only about 3,000 have been used as regular crops, and approximately 150 are cultivated commercially [17]. Wild plants are consumed not only during periods of food scarcity but also during times of ecological abundance, indicating their persistent relevance in human diets [17, 74]. Globally, more than 300 million people still rely on forest resources for part of their nutrition and livelihood security [11], and the dietary and health benefits of wild foods have been well documented across diverse ecological regions [1, 10, 21, 23, 40].

In Pakistan, ethnobotanical research has traditionally focused on medicinal flora, resulting in limited scientific attention toward wild edible plants [12]. Pakistan spans 796,000 km², encompassing a wide range of altitudes (from sea level to 8,611 m) and ecological zones from coastal areas to high mountains supporting over 6,000 vascular plant species [63, 64, 66]. The country ranks among the most food-insecure regions globally, with approximately 40% of households categorized as diet-insecure, especially in the northwestern districts [30]. Rapid population growth, combined with recurrent natural and anthropogenic disturbances, has intensified pressure on available food resources [60, 61]. This highlights a growing need to identify sustainable and locally adapted alternative food sources. Previous work in northwest Pakistan has documented the widespread use of wild vegetables among rural communities, emphasizing their cultural and nutritional significance [7].

Food insecurity affects both developing and developed nations, and nutrient deficiencies remain widespread. In Pakistan, hunger-related mortality among adolescents is a significant concern [72], underscoring the importance of exploring non-cultivated food resources. Despite their potential, the role of wild plants in contributing to nutrition and food security in Pakistan remains insufficiently studied. Research from Swat Valley indicates that wild plant trade provides supplementary income for rural households, though concerns regarding overharvesting and sustainability persist [13]. Ethnobotanical surveys from neighboring regions such as Utror and Gabral have documented extensive traditional knowledge associated with wild edible and medicinal plants [31], while earlier studies in Swat have reported diverse cultural uses of wild flora and substantial local ecological knowledge [32]. Similar patterns of traditional plant use and knowledge transmission have been observed in the Kurram region [35], Kabal Valley [39], and Manrai Hills [38], where the botanical richness of the region has been emphasized. Studies from Sargodha further indicate that many wild vegetables provide important nutritional and medicinal benefits [40]. Research from Dir Kohistan supports the widespread reliance on wild plants among mountain communities [41], while ethnobotanical assessments from Charbagh (Swat) reveal a rich cultural heritage of plant use consistent with findings across the region [43]. Additional studies from Shogran Valley in northern Pakistan confirm that local communities maintain extensive traditional practices involving herbs, shrubs, and trees, underscoring the national significance of wild flora [48].

To address these gaps, this study adopts a comparative and analytical approach, with the following hypotheses:

1. Diversity and use of wild edible plants vary across ecological zones and altitudinal gradients, reflecting cross-geographical differences in plant availability and utilization.
2. Traditional ecological knowledge differs among ethnic and linguistic groups, highlighting cross-cultural variation in plant uses, nomenclature, and management practices.
3. Certain plants contribute disproportionately to local diets and cultural practices, indicating their critical role in nutrition, food security, and cultural heritage.

Based on these hypotheses, the study aims to document the diversity of wild edible plants, evaluate associated traditional knowledge, assess their nutritional contributions, compare findings across ecological zones and ethnic groups, and provide baseline data to support conservation, sustainable use, and policy interventions in Swat Valley.

## Materials and methods

### Study area and village selection

**Fig. 1:**
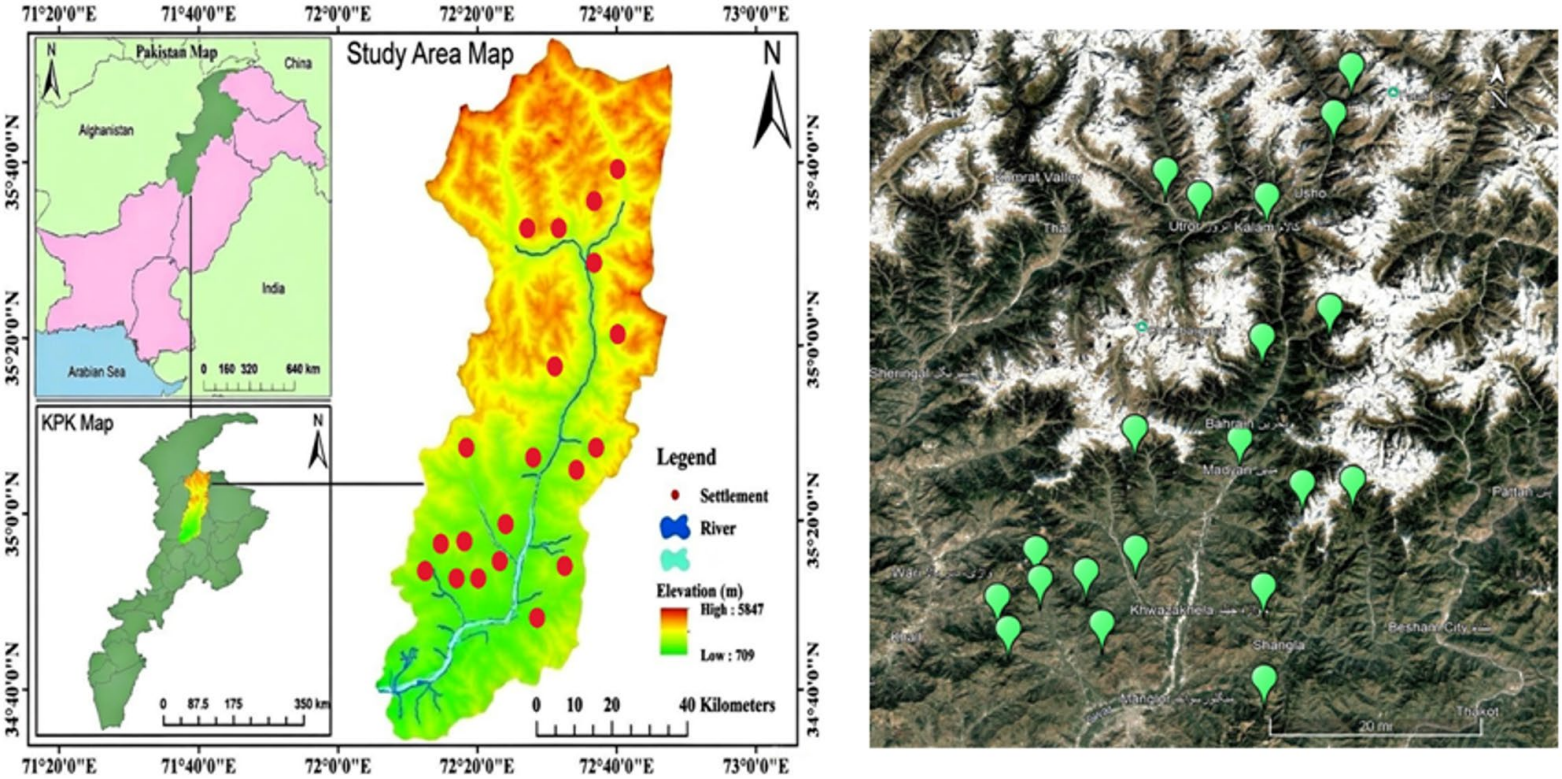
Map of study area

The study area lies in the remote Hindu Kush region of Pakistan, at the junction of three major mountain ranges: the Karakoram, Hindukush, and Himalayas [6] (Fig. 1). It is among the most scenic regions in Asia and is often referred to as the “Switzerland of Asia” [14, 16, 17, 45, 73]. Swat is renowned for its beautiful valleys and archaeological remains of the ancient Gandhara civilization. Geographically, it is located between 34°34′ to 35°55′ N and 72°08′ to 72°50′ E [44]. A total of 20 villages across six tehsils were selected for data collection based on their traditional and cultural significance. These include seven villages from Bahrain Tehsil, four from Kabal, four from Matta, two from Charbagh, two from Khwazakhela, and one from Madyan Tehsil. The selected communities represent diverse cultural traditions, allowing the study to encompass a wide range of ethnobotanical knowledge and practices. Each village was cross-checked with others within and across tehsils to ensure the accuracy, reliability, and comprehensiveness of the collected information. This distribution reflects the concentration of sampling in Bahrain and the relative sparsity in Madyan, emphasizing the importance of accounting for tehsil-specific ecological and cultural variations.

The studied villages span a broad altitudinal range from 600 m in Gulibagh to 5,957 m in Mankyal covering low valleys, mid-elevation areas, and high mountain zones. Altitude strongly influences plant diversity, phenological patterns, and availability, thereby affecting local ethnobotanical knowledge and resource use. Climatic conditions vary significantly across the area, with average annual rainfall ranging from 524 mm in Gabral to 3,310 mm in Utror, and mean temperatures between 4 °C in Gabral and 26 °C in Derdyal. Such variation creates distinct ecological niches that shape both plant distribution and traditional utilization patterns. Villages in Tehsil Bahrain (Madyan, Mankyal, Bahrain, Gabral, Kalam, Mahodand, Usho, and Utror) include both high- and mid-elevation zones. Tehsil Kabal (Asharai, Derdyal, Manarai Godha, and Qalagai) mainly covers mid- to high-elevation areas, while Tehsil Matta (Churprial, Darmai, PirKalay, and Shawar) lies at mid-altitudes. Charbagh (Gulibagh and Malam Jabba) and Khwazakhela (Fatehpur and Miandam) encompass moderate to high elevations with a temperate climate. This diverse environmental and cultural matrix provided a comprehensive framework for ethnobotanical documentation. The inclusion of cross-verified data from multiple villages ensures reliable representation of both local and regional variations in plant use and ethnobotanical understanding.

The Swat District is bordered by Chitral and Ghizer in the north, Indus Kohistan and Shangla in the east, Buner and Malakand in the south, and Dir in the west [31, 43, 44]. It covers an area of approximately 5,337 km² and has a population of about 2.3 million, increasing from 1.26 million in 1999. The district is divided into upper and lower Swat, both characterized by distinct climatic and topographical features. Annual rainfall ranges between 500 and 1,200 mm, with snowfall of 423.56–600 cm [6]. Upper Swat is mountainous, snow-covered for most of the year, while lower Swat is relatively flat, densely populated, and experiences little to no snowfall. Climatic variability in Swat, including snowfall patterns and meltwater dynamics, has been shown to strongly influence vegetation distribution in the region [26].

Winters are cold, with temperatures dropping to 2.4 °C from December to February, whereas summers can reach up to 36.3 °C [6]. The region experiences two monsoon cycles, winter and summer [24], and is vulnerable to climatic variability and biodiversity loss June is typically the hottest month (16–33 °C), while January remains the coldest [65]. Summer monsoon rains [11] support cultivation of two crops in lower zones and a single crop at higher elevations.The area’s unique climatic and topographical conditions foster a rich and diverse flora [6], making it both an ecological hotspot and a major tourist destination [44]. The district’s vegetation ranges from tropical dry deciduous forests to alpine grasslands, hosting approximately 1,550 vascular plant species and 55 pteridophyte species [6]. Among these, 345 species are reported to possess medicinal value.

### Ethnic and socioeconomic profile of swat Valley communities

District Swat is home to a rich mosaic of cultural communities, reflecting diverse ethnicities, languages, and ecological adaptations within the Hindu-Kush mountain range [45]. The region’s population relies heavily on indigenous food resources, with wild edible plants (WEPs) collected from both mountainous and agricultural landscapes. Traditional cooking methods are commonly employed by local households, particularly by women, to prepare these plants in ways that are both nutritious and palatable [9, 31]. The majority of the population consists of Pashto-speaking Yousafzai Pathans, who primarily inhabit lower-altitude and temperate forest areas such as Bahrain, Charbagh, Kabal, Matta, and Madyan tehsils. These communities utilize wild edible plants alongside cultivated crops, dairy products, and livestock, forming an integral part of their semi-subsistence food system [18].

In contrast, the northern high-altitude and alpine regions such as Mankyal, Mahodand, Ushu, Kalam, Utror, and Gabral are predominantly inhabited by Kohistani, Torwali, Kalami, and Gujjar communities. These groups maintain distinct linguistic identities, speaking dialects such as Kohistani, Gojri, Kalami, and Torwali [9, 31, 59]. Their livelihoods are closely tied to the alpine and subalpine environments, where they engage in gathering WEPs during grazing, hunting, and fuelwood collection, while keeping small herds of sheep, goats, cows, and buffaloes for dairy needs.

Field surveys were conducted in 20 villages across six tehsils, spanning a wide range of elevations (1,062–3,012 m) and ecological zones, including riverine forests, temperate forests, alpine forests, and alpine meadows. The study involved 144 participants, with both male and female informants, primarily aged 50–90 years, highlighting the pivotal role of elders in the transmission of traditional ecological knowledge. Notably, the composition of participants varied across villages, with higher numbers of informants in alpine villages such as Mankyal (26 participants) and Kalam (21 participants), reflecting the richness of local knowledge in high-altitude communities (Table 1).

This diverse ethnic and linguistic composition, coupled with ecological heterogeneity, has fostered unique traditional practices for the collection, preparation, and use of wild edible plants. Such practices not only contribute to household nutrition and food security but also serve as a repository of cultural identity, ecological adaptation, and intergenerational knowledge transfer [9, 18, 31, 59].

Table 1. Summarize data reflect the diversity of communities engaged in traditional collection and use of wild edible plants, highlighting both cultural and ecological variations across the Swat Valley.

**Table 1:** Demographic, Ethnolinguistic, and ecological characteristics of study participants across selected villages in district Swat, Pakistan

Tehsil	Village	Latitude (°*N*)	Longitude (°E)	Elevation (m)	Ecology	Ethnicity	Language(s)	Respondents (*n*)	Gender/Age
Bahrain	Mankyal	35.3284	72.6181	2000	Alpine Meadow	Kohistani and Gujjar	Kohistani, Gujjaro	13	10 M/5 F; majority 50–90
Bahrain	Bahrain	35.2072	72.5456	1425	Temperate Forest	Yousafzai, Gujjar and Kohistani	Poshto, Gujjaro and Kohistani	16	11 M/6 F; mixed ages
Bahrain	Gabral	35.525	72.4125	2286	Riverine and Forest	Kohistani and Gujjar	Kohistani and Gujjaro	9	6 M/3 F; majority 50–90
Bahrain	Kalam	35.4902	72.5796	2001	Temperate Forest	Kohistani, Gujjar, Torwali	Kohistani Gujjaro, Torwali	12	8 M/5 F; majority 50–90
Bahrain	Mahodand	35.7139	72.651	2865	Alpine Forest	Torwali and Gujjar	Torwali and Gujjaro	6	4 M/2 F; majority 50–90
Bahrain	Ushu	35.6076	72.6902	2300	Alpine Meadow	Torwali and Gujjar	Torwali and Gujjaro	11	9 M/2 F; majority 50–90
Bahrain	Utror	35.491	72.4687	2300	Temperate Forest	Kalami, Kohistani, Torwali and Gujjar	Kalami, Kohistani, Torwali, Gujjaro	14	10 M/6 F; majority 50–90
Charbagh	Gulibagh	34.8803	72.4554	1050	Riverine and Forest	Yousufzai	Pashto	7	5 M/3 F; majority 50–90
Charbagh	Malam Jabba	34.7999	72.5722	2465	Alpine Forest	Yousufzai, Torwali and Gujjar	Pashto, Torwali and Gujjaro	6	4 M/2 F; majority 50–90
Kabal	Asharai	34.6873	72.7135	1138	Temperate Forest	Gujjar	Gujjaro	10	7 M/3 F; majority 50–90
Kabal	Derdyal	34.9368	72.206	1687	Temperate Forest	Yousufzai	Pashto	10	5 M/5 F; majority 50–90
Kabal	Manarai Godha	34.98	72.2	3012	Alpine Forest	Kohistani, Gujjaro and Torwali	Kohistani, Torwali and Gujjaro	4	2 M/2 F; majority 50–90
Kabal	Qalagai	34.7938	71.7741	1288	Temperate Forest	Gujjar, Shephered and Yousufzai	Pashto and Gujjaro	5	3 M/2 F; majority 50–90
Khowazakhela	Fatehpur	35.0678	72.4865	1687	Temperate Forest	Yousufzai	Pashto	5	3 M/2 F; majority 50–90
Khowazakhela	Miandam	35.0541	72.5648	1880	Alpine Meadow	Yousufzai, Kohistani and Gujjar	Poshto, Kohistani Gujjaro	6	4 M/2 F; majority 50–90
Matta	Churprial	34.9811	72.3632	1288	Temperate Forest	Pashtun an d Gujjar	Pashto and Gujjaro	5	3 M/2 F; majority 50–90
Matta	Darmai	35.0814	72.4473	1423	Temperate Forest	Yousufzai	Pashto	5	3 M/2 F; majority 50–90
Matta	PirKalay	34.1295	71.5034	1062	Temperate Forest	Yousufzai and Gujjar	Pashto and Gujjaro	4	2 M/2 F; majority 50–90
Matta	Shawar	35.4309	74.6357	1548	Temperate Forest	Yousufzai	Pashto	5	3 M/2 F; majority 50–90
Bahrain	Madyan	35.1404	72.5353	1320	Temperate Forest	Yousufzai	Pashto	7	5 M/2 F; mixed ages

### Data collection

The present study was conducted from 2018 to 2022 across various localities of Swat District, Pakistan, to document the diversity and traditional uses of wild edible plants. Data were collected from well-informed participants, including herb sellers, shepherds (locally called *Shponkay*), Gujjars, and traditional herbalists, all of whom possess extensive knowledge of local plant resources. A total of 160 informants (100 men and 60 women), aged between 20 and 90 years, were engaged in the study. The Informants were selected using purposive and snowball sampling methods to ensure inclusion of individuals with significant traditional knowledge. Special attention was given to age, gender, occupation, and residence to capture diverse perspectives. Elderly members and long-term residents were prioritized as they often hold comprehensive knowledge of wild edible plants and their traditional uses. The Data were collected using a semi-structured questionnaire, which included both open- and close-ended questions. The questionnaire focused on: (i) Local names of plants and plant parts used, (ii) Methods of collection, preparation, and consumption, (iii) Seasonal availability and frequency of use (iv) Nutritional and medicinal properties as perceived by informants (v) Cultural significance and traditional knowledge transmission. In addition to questionnaires, in-depth interviews and personal observations were conducted to validate the information and capture additional details that may not be listed in the structured questions. During field surveys, plant specimens were carefully collected, pressed, and dried following standard herbarium protocols. All collected plant specimens were assigned unique field numbers for accurate documentation and tracking. Species identification was carried out using the *Flora of Pakistan* online database and related printed volumes (Nasir & Ali 1970–1979; Nasir & Ali 1980–1989; Ali & Nasir 1989–1992; Ali and Qaisar 1996–2006; Ali & Qaiser 1993–2009). Botanical names of all recorded species were further verified and updated according to the World Flora Online database (http://www.worldfloraonline.org/), ensuring nomenclatural accuracy, standardization, and consistency with global taxonomic references. This systematic approach, combining purposive informant selection, a carefully structured questionnaire, and rigorous field verification, enabled the accurate documentation of plant diversity, traditional uses, and the preparation of a reliable, annotated inventory of wild edible plants in the Swat region.

### Cultural significance and use value indices

#### Frequency of citation (FC)

Frequency of Citation (FC) represents the number of informants who mentioned a particular plant species in an ethnobotanical study. It reflects how commonly a plant is known or used within a community.

$$\:FC=Number\:of\:informants\:who\:mentioned\:the\:species$$

#### Use reports (UR)

Use Reports (UR) indicate the total number of times a plant species was reported for any specific use, which can include multiple uses by the same informant. UR helps to show the diversity of uses for a plant.

$$\:UR=\sum\:All\:uses\:reported\:by\:all\:informants\:for\:a\:species$$

#### Use value (UV)

Use Value (UV) is a quantitative measure of the relative importance of a species. It considers how many uses each informant reports for a species and averages it over all informants. A higher UV indicates greater cultural significance.

$$\:UV=\sum\:Ui/n$$

Where *Ui​* = number of uses mentioned by the informant for a species and *n* = total number of informants.

### Relative frequency of citation

Relative Frequency of Citation (RFC) shows how frequently a plant is mentioned by informants relative to the total number of informants. It ranges from 0 (no one mentioned the plant) to 1 (everyone mentioned it), helping to identify culturally important plants.

$$\:RFC=FC/N$$

Where *FC* = frequency of citation and *N* = total number of informants.

### Jaccard index (JI)

The Jaccard Index is a statistical measure used to quantify the similarity between two sets. It’s widely applied in ecology, bioinformatics, text analysis, and ethnobotany to compare shared elements between datasets.

For two sets A and B:

$$\:J\left(A,B\right)=\frac{\mid\:\mathrm{A}\cap\:\mathrm{B}\mid\:\:}{\mid\:\mathrm{A}\cup\:\mathrm{B}\mid\:\:}$$

Where: ∣A∩B∣ = number of elements common to both sets and ∣A∪B∣ = total number of elements in either set (union). The Jaccard Index ranges from 0 to 1: 0 → no common elements and 1 → sets are identical.

### Chi-square

To assess whether the observed distribution of plant use overlap differed from expected values, a Chi-square (χ²) goodness-of-fit test was conducted. Observed counts (O) were compared with expected counts (EEE), calculated under the assumption of a uniform distribution. The Chi-square statistic was calculated

$$\:\boldsymbol{\chi\:}2=\sum\:\frac{(\boldsymbol{O}\boldsymbol{i}\boldsymbol{}-\boldsymbol{E}\boldsymbol{i})2}{\boldsymbol{E}\boldsymbol{i}}$$

Where: χ2 = Chi-square test statistic, *Oi​* = observed frequency in category *i*,* Ei​* = expected frequency in category *i*,* ∑ =* summation over all categories. Degrees of freedom were determined as: *df*= (number of categories − 1). A *p-value < 0.05* was considered statistically significant. Data were organized in Microsoft Excel, and statistical analyses were performed using R software.

## Results

### Demographic characteristics of study participants

A total of 160 informants participated in the study, including 100 males (62.5%) and 60 females (37.5%) Table 2. The higher participation of men reflects local sociocultural patterns, where men are more involved in farming, herding, and outdoor plant collection, while women play a central role in food preparation and related knowledge. Most informants were older adults with 56.25% aged 50 to 90 years and 31.25% aged 30 to 50 years indicating that traditional knowledge of wild edible plants is primarily retained among elders. The majority of participants were illiterate (71.8%) while 18.75% had school-level education and 9.37% had university level education suggesting that ethnobotanical knowledge is largely transmitted orally and through daily livelihood practices rather than formal schooling. In terms of occupation farmers (43.75%), laborers (31.25%), and shepherds (18.75%) were the dominant groups highlighting that individuals directly engaged in agriculture and livestock management have the greatest interaction with local flora and contribute most substantially to traditional plant knowledge.

**Table 2:** Demographic characteristics of the study participants

Demographic characteristics	Number	Percentage %
**Sex**		
Male	100	62.5
Female	60	37.5
**Age**		
16–30	20	12.5
30–50	50	31.25
50–90	90	56.25
**Education**		
School	30	18.75
University	15	9.37
Uneducated	115	71.8
**Profession**		
Teacher	10	6.25
Farmer	70	43.75
Shephard	30	18.75
Laborers	50	31.25

### Diversity of wild edible plants and cultural significance

The present study documented a total of 175 wild edible plant species belonging to 72 families from the Swat Valley. These species are deeply embedded in the traditional diet of local communities, serving not only as vegetables, fruits, flavoring agents, and beverages but also as markers of cultural identity and local culinary heritage. Herbs were the most dominant growth form (62.3%), followed by shrubs (20.6%), trees (16.0%), and climbers (1.1%) (Table 3), reflecting the community’s preference for easily accessible, fast-growing vegetation. Young shoots or stems were the most frequently utilized plant parts (41.7%), emphasizing their accessibility and seasonal importance, particularly in spring and summer when cultivated vegetables are scarce. Fruits accounted for 30.6% of uses, while leaves were used in 13.9% of preparations such as salads, soups, and cooked vegetables. Other plant parts seeds (5.6%), flowers (3.3%), pods (2.8%), whole plants (1.7%), and bark (0.6%) though less commonly used, contribute to dietary diversity and reflect culturally specific culinary practices. For example, flowers and pods are often incorporated into traditional recipes or used as flavoring agents, demonstrating a sophisticated local understanding of plant properties and taste preferences.

The use value (UV) and relative frequency of citation (RFC) highlight culturally important species, with *Berberis lycium* showing the highest UV (0.813) and RFC (0.75) indicating its widespread recognition and daily integration into local diets (Table 4). Other commonly consumed species include *Chenopodium album* UV (0.68), RFC (0.63), *Berberis vulgaris* UV (0.65), RFC (0.63), *Abies pindrow* UV (0.563), RFC (0.50), *Amaranthus graecizans* UV (0.46), RFC (0.44), *Amaranthus spinosus* UV (0.43), RFC (0.41), *Amaranthus viridis* UV (0.40, RFC (0.38), *Asparagus officinalis* UV (0.37), RFC (0.34), *Brassica juncea* UV (0.37), RFC (0.34), and *Cichorium intybus* UV (0.37), RFC (0.34) (Table 7). These species are not only nutritionally important but also carry social and cultural value, often associated with customary dishes, seasonal rituals, and market trade.

The gradient of UV 0.113 to 0.813) and RFC 0.09–0.75 values illustrates a spectrum of cultural significance and frequency of use. While a few species are highly preferred and culturally prominent the majority are recognized and consumed less frequently, reflecting the dynamic, unevenly distributed traditional knowledge base of the Swat Valley. Local participants’ extensive contributions underscore the intergenerational transmission of ethnobotanical knowledge demonstrating how plant use practices are shaped by ecological availability, seasonal cycles, and culturally embedded culinary traditions.

**Table 3:** Wild food plant diversity Habit, plant parts Used, gathering Period, and traditional food uses

Category	Sub-category	Count	Total %
**Habit**	Herb	110	62.9%
	Shrub	35	20%
	Tree	27	15.4%
	Climber	3	1.7%
**Used part**	Young shoot	73	41.7%
	Leaves	23	13.1%
	Fruits	52	29.7%
	Seed	9	5.1%
	Pods	5	2.9%
	Flowers	4	2.3%
	Bark	1	0.6%
	Whole plant	1	0.6%
**Gathering period**	Winter	14	8%
	Spring	23	13.1%
	Summer	130	74.3%
	Any season	5	2.9%
	Multiple seasons	3	1.7%
**Traditional food use**	Fruits	69	39.4%
	Vegetables	65	37.1%
	Raw	25	14.3%
	Cooked	8	4.6%
	Tea/Flavoring	9	5.1%
	Oil	4	2.3%
	Salad	9	5.1%
	Curry	1	0.6%
	Sauce/Chutney	2	1.1%
**Plant species**	Total species	175	100%
**Family**	Total families	72	-

### Diversity and traditional uses of wild edible plants in swat district

Swat District harbors a rich diversity of wild edible plants that form an essential component of the local diet and traditional practices. These species are utilized in multiple ways including as vegetables, fruits, sauces, herbal teas and flavoring agents reflecting deep-rooted ethnobotanical knowledge transmitted across generations. The collection and use of these plants not only provide important nutritional resources but also support local livelihoods and contribute to the preservation of cultural heritage. Their integration into daily diets and customary culinary practices highlights the dynamic relationship between the local communities and their natural environment.

### Vegetable species

**Fig. 2:**
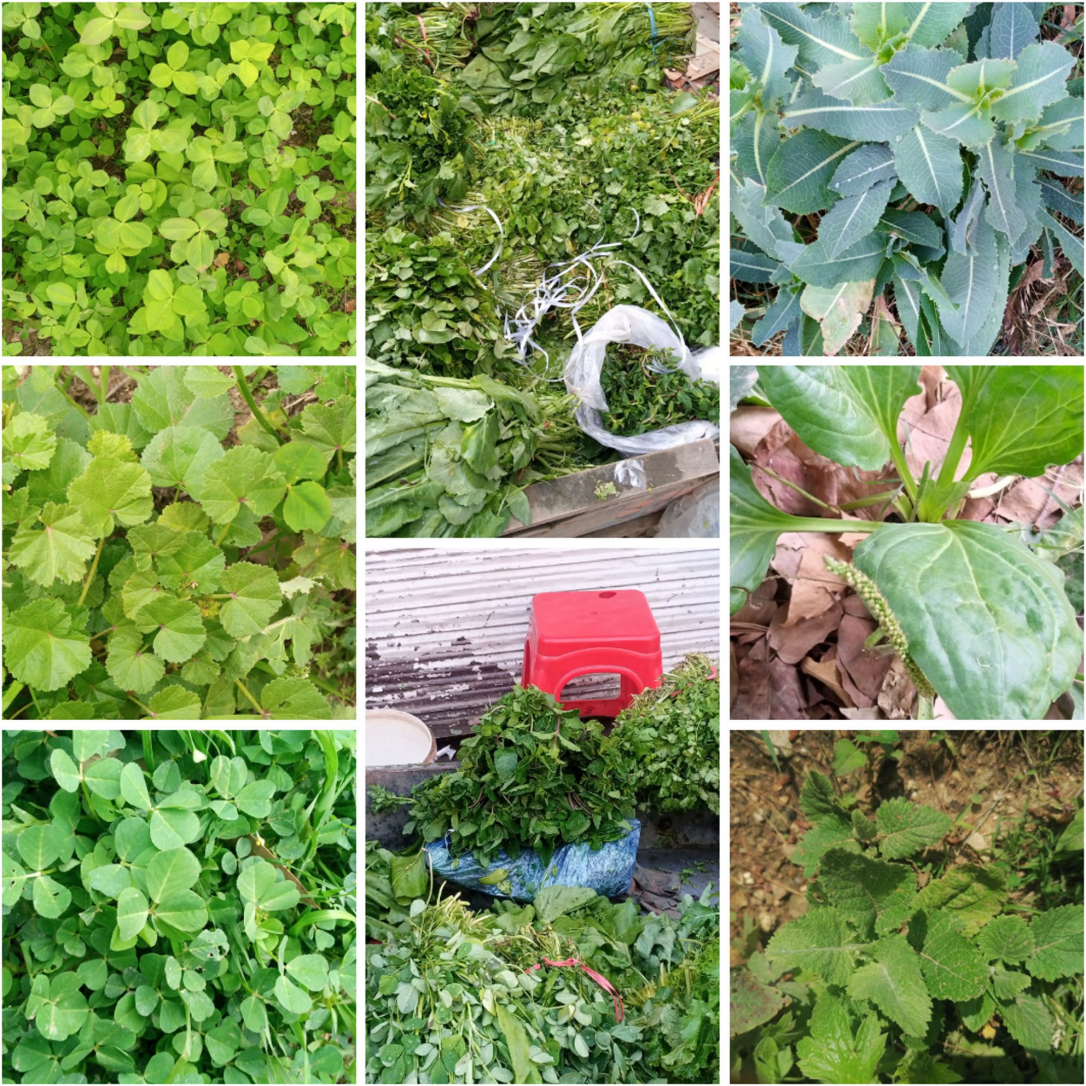
Some wild edible vegetable plants; *Trifolium repensm*,* Nasturtium officinale*,* Lactuca abietina*,* Malva neglecta*,* Cardamine hirsute*,* Plantago ovate*,* Medicago polymorpha*,* Urtica urens*

In the present study, approximately 37% of documented wild edible plants were used as vegetables (Tables 3 and 7). The most frequently cited species included *Nasturtium officinale*, *Trifolium repens*, *Dryopteris juxtapostia*, *Amaranthus viridis*, *Amaranthus spinosus*, *Brassica rapa*, *Chenopodium album*, *Medicago polymorpha*, *Portulaca oleracea*, *Portulaca quadrifida*, *Malva neglecta*, and *Cichorium intybus*. Traditional knowledge regarding their use is transmitted orally across generations and is deeply embedded in daily life. Typically, local people collect young shoots and leaves as shown in (Fig. 2), clean them and prepare them using customary cooking methods. Fresh leaves and shoots are often chopped, boiled and combined with garlic, tomatoes, chilies, and oil to prepare a traditional mixed dish locally known as “taarka,” reflecting the integration of ecological knowledge into culinary practice.

Among these vegetables, *Nasturtium officinale* (locally “Tarmeera”) is highly valued thriving in marshy areas during summer and is also used medicinally to treat stomach ailments. *Trifolium repens* which emerges primarily in winter and is frequently collected by women for food preparation highlighting their central role in maintaining dietary traditions. *Amaranthus viridis* grows alongside maize during summer while *Dryopteris juxtapostia* is prized in “sag” dishes sold in local markets. *Amaranthus spinosus* serves both as a vegetable and cattle fodder, believed to enhance milk production demonstrating the interconnectedness of dietary and livestock practices. *Brassica rapa* is a versatile species whose leaves, seeds, and roots are consumed as food, used for animal feed, and whose oil is highly valued. *Chenopodium album* is an annual summer plant prepared as a stewed vegetable, while *Medicago polymorpha*, growing in old meadows, is primarily consumed in spring and winter. *Portulaca oleracea* and *P. quadrifida* are commonly added to yogurt-based dishes in remote regions, *Malva neglecta* is harvested from cemeteries and old meadows during winter, primarily by economically disadvantaged families, and *Cichorium intybus* occurs in fields during spring and summer, consumed both as a vegetable and in salads.

The study also documented culturally unique culinary preparations. “Chokaner” (also called “warjali”) is a traditional mixed vegetable dish in which women gather and chop *Trifolium repens*, *Asphodelus tenuifolius*, *Euphorbia malaica*, *Rumex dentatus*, *Medicago polymorpha*, *Sisymbrium altissimum*, and *Carthamus oxyacantha*, then boil the mixture with garlic and coriander, adding rice to create a highly appreciated dish. Similarly, young shoots of *Asparagus* spp. are collected and cooked with milk, eggs, or meat (“qeema”), commanding high market value due to their distinctive flavor.

However, wild edible plants play a critical role in local diets, particularly for economically disadvantaged families. Seasonal collection from meadows, marshlands, and cemeteries not only provides essential nutrition but also income through local markets. Women are central to gathering, cooking, and transmitting traditional knowledge, emphasizing the cultural and intellectual heritage embedded in local culinary practices.

### Fruit species

**Fig. 3:**
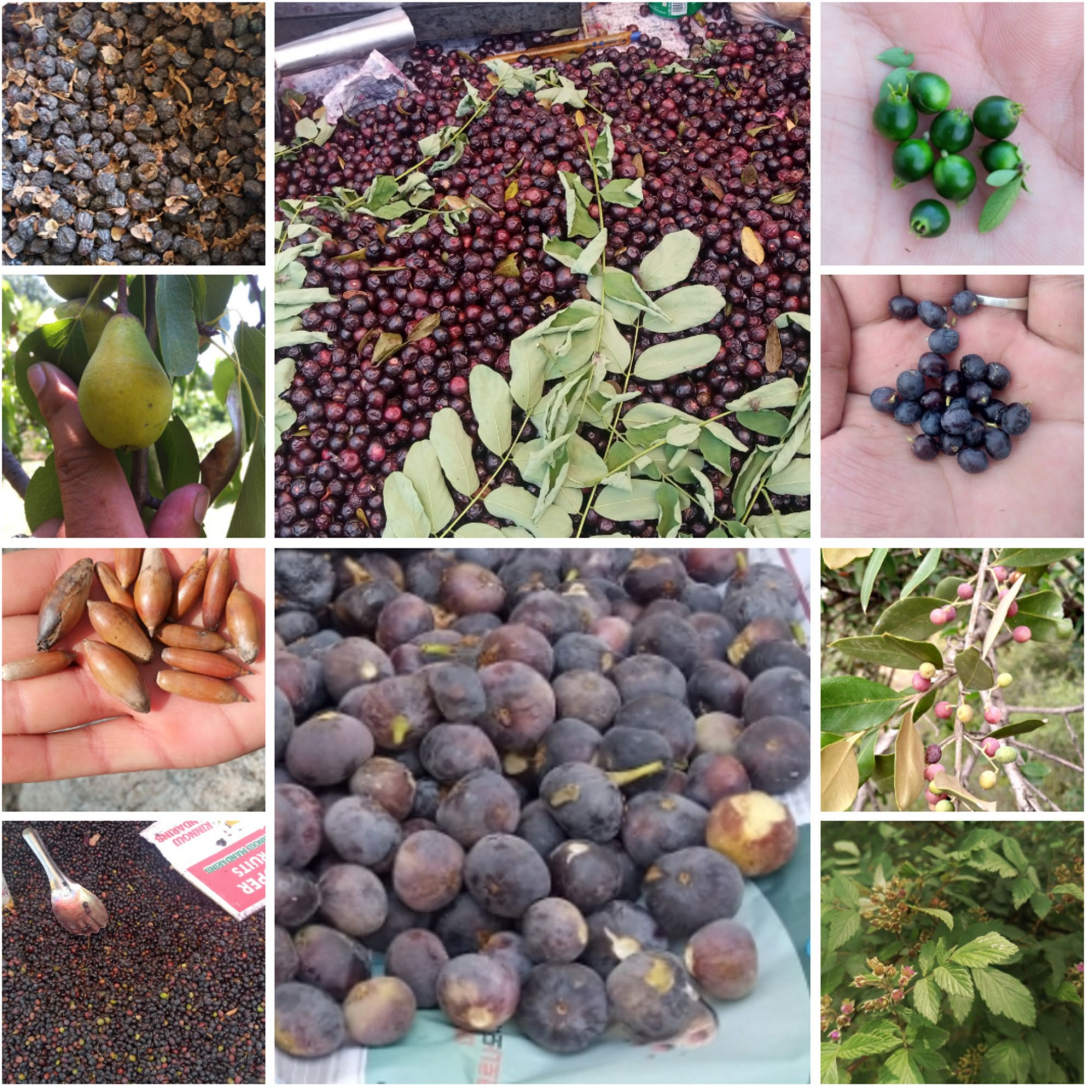
Some wild edible fruits of the area; Diospyros lotus, *Monotheca sideroxylon*,* Mysine Africana*, *Myrtus communis*,* Phyrus spp*,* Quercus dilatata*,* Ficus palmate*,* Olea ferruginea*,* Berberis lyceum*,* Rubus ellipticus*

In the present study, about (39%) of the documented wild edible plants were used as fruits (Table 7). Local communities collected these fruits from their natural habitats, predominantly consuming them fresh reflecting both nutritional and cultural significance as shown in (Fig. 3). The most commonly reported wild fruits included *Ficus carica*, *Berberis lyceum*, *Myrtus communis*, *Olea ferruginea*, *Diospyros lotus*, *Elaeagnus umbellata*, *Ficus palmata*, *Morus alba*, *Morus nigra*, *Morus laevigata*, *Sideroxylon mascatense*, *Punica granatum*, *Zizyphus oxyphylla*, *Zizyphus sativa*, *Zizyphus jujuba*, *Fragaria indica*, and *Rubus fruticosus*. Among these, *Ficus palmata*, *Ficus carica*, *Morus alba*, *Morus nigra*, and *Morus laevigata* were particularly abundant and widely consumed.

Fruits were generally harvested during cooler parts of the day morning or evening to avoid the intense summer heat, particularly in June and July. *Berberis lyceum* was collected earlier in May and June with its juice also consumed as a refreshing and cooling beverage. *Sideroxylon mascatense* occurring at lower mountain elevations, was carefully harvested in large containers to prevent spoilage, while *Diospyros lotus* fruits were frequently dried for off-season use or local market trade a common practice in hillside areas.

Children often consumed *Olea ferruginea* fruits directly in the fields during summer highlighting the integration of wild fruits into daily life and informal dietary practices. *Elaeagnus umbellata* provided ruby-colored fruits valued both as food and in traditional remedies being rich in carotenoids such as lycopene. *Punica granatum* is widely cultivated and prized for its economic and nutritional value. Fruits of *Zizyphus oxyphylla*, *Z. jujuba*, and *Z. sativa* were collected fresh or dried for off-season use and also incorporated in traditional medicine. Slightly sour *Fragaria indica* fruits were commonly eaten by children as a heart tonic while *Rubus fruticosus* fruits were gathered throughout summer for both consumption and medicinal purposes. *Pyrus pashia* fruits were collected in early winter and consumed raw. Additionally, *Myrtus communis* produced palatable fruits and its dried flowers, leaves and fruits were traditionally used to flavor foods during summer.

Although, wild fruits constitute a vital component of the local diet especially for economically disadvantaged families. Their collection and consumption not only supplement seasonal food supplies and provide essential nutrients but also reinforce cultural food practices maintain dietary diversity, and reflect the Swat Valley communities’ deep ecological knowledge and relationship with their natural environment.

### Sauce and chutneys

Wild plants in the study area are commonly used to prepare sauces and chutneys with approximately 4 species (2%) reported for this purpose including *Mentha royleana*, *M. arvensis*, *Rumex hastatus*, and *Zanthoxylum armatum*. During field surveys local informants shared detailed preparation methods such as tomatoes and *Mentha* leaves were typically crushed and combined with dried powder of *Zanthoxylum armatum* seeds and occasionally lemon juice was added to enhance flavor. In some cases the ingredients were mixed with water to create a sauce. These herbal sauces are highly popular within the local communities and people often dry the plants during their respective seasons for off-season use. Both fresh and dried forms are employed depending on availability. The study findings highlight that the preparation and use of wild plants for sauces and chutneys vary across regions, reflecting localized traditional knowledge and culinary practices.

### Species used fresh or raw

Approximately 25 wild plant species (14% of the documented flora) were consumed raw highlighting a significant aspect of foraging-based dietary practices (Table 7). Collection and consumption of these plants were particularly common among children, who often chewed fruits, seeds, or shoots directly from the wild for refreshment, flavor, or thirst-quenching purposes. Prominent examples include *Pinus gerardiana*, *Rhus chinensis*, *Eryngium caeruleum*, *Scandix pecten-veneris*, *Onopordum acanthium*, *Onosma hispida*, *Lathyrus aphaca*, *Lathyrus sativus*, *Vicia monantha*, *Vicia tetrasperma*, and *Vicia hirsuta*.

This practice reflects a deep-rooted cultural relationship with the local environment, where children’s early engagement with wild plants fosters traditional ecological knowledge from a young age. Eating plants raw is not only a practical strategy for immediate nutrition but also a culturally informed behavior, often tied to seasonal availability and the palatability of specific species. These raw consumption habits contribute to dietary diversity help introduce young members of the community to wild edibles, and play a role in the transmission of ethnobotanical knowledge across generations.

### Wild food plants used in herbal teas and as flavoring agents

Approximately 5% of the documented wild plant species were used as flavoring agents or herbal teas (Table 7) reflecting the local community’s nuanced knowledge of plant properties. Among these, the leaves of *Myrtus communis*, *Abies pindrow*, *Thymus serpyllum*, *Oenothera rosea* and *Cymbopogon citratus* were most commonly utilized, with *Myrtus communis* and *Cymbopogon citratus* being particularly widespread. These plants were valued not only for their aroma and taste but also for their medicinal and refreshing properties. Additionally, species, including *Foeniculum vulgare*, *Mentha longifolia*, *Mentha arvensis*, and *Zanthoxylum alatum*, were employed primarily as flavoring agents to enhance daily meals or beverages. The leaves of *Myrtus communis* and *Cymbopogon citratus* were often dried and steeped to prepare herbal teas with the tea of *Cymbopogon citratus* holding a special place in marriage ceremonies and other social gatherings symbolizing hospitality and celebration.

These practices illustrate a strong cultural connection between plant use and social traditions. Herbal teas and flavoring agents are not consumed solely for nutrition or taste; they are embedded in communal rituals, seasonal celebrations, and health practices. Children, women, and elders all contribute to the gathering, preparation, and consumption of these plants, highlighting the transmission of traditional ecological knowledge across generations. Moreover, the preference for aromatic and medicinal species reflects a culturally mediated understanding of plant properties, where sensory experience, therapeutic effects, and symbolic meanings intertwine in everyday life.

### Marketing of wild edible plants

Wild edible plants in Swat Valley not only serve as dietary resources but also provide an important source of income for local communities. About 27 plant species (15% of documented taxa) were traded in local markets, with prices influenced by seasonal availability, quality, and demand (Tables 4 and 7). Vegetables such as *Dryopteris odontoloma*, *Dryopteris juxtapostia*, *Amaranthus viridis*, *Daucus carota*, *Caralluma tuberculata*, *Caralluma edulis*, *Carthamus oxyacantha*, *Nasturtium officinale*, *Brassica rapa*, *Brassica juncea*, *Trifolium repens*, *Malva sylvestris*, *Portulaca oleracea*, and *Mentha royleana* were commonly sold as fresh produce. Several fruits, including *Berberis lyceum*, *Diospyros lotus*, *Morus alba*, *Ficus palmata*, *Morus laevigata*, *Zizyphus mauritiana*, *Ziziphus jujuba*, *Monotheca buxifolia*, and *Vitis vinifera*, were also commercially important. While most were sold fresh, some species such as *Ficus palmata*, *Morus laevigata*, *Vitis vinifera*, *Diospyros lotus*, and *Ziziphus jujube* were marketed in dried form, extending their shelf life and trade potential.

The sale of wild edible plants reflects a culturally embedded practice that links ecological knowledge with livelihoods. Local women and men often participate in the collection and marketing of these plants, with women particularly involved in harvesting, processing, and preparing vegetables and herbs for sale. This trade not only supplements household income but also reinforces the transmission of traditional plant knowledge, as market interactions require accurate identification, understanding of seasonal availability, and culinary expertise. Moreover, marketing of wild foods demonstrates the adaptive strategies of communities in Swat, where reliance on both cultivated and wild resources ensures food security and economic resilience, particularly for poorer families or those in remote areas.

**Table 4:** Wild food plants marketing

Species	Form	Price range ($)	Season of availability
*Amaranthusviridis*	Fresh	0.26	Jun-Aug (Summer)
*Berberis lyceum*	Fresh and dried	1.74	May-Jun (Summer)
*Brassica juncea*	Fresh	0.22	Dec-March (Winter)
*Brassica rapa*	Fresh	0.26	Dec-March (Winter)
*Caralluma edulis*	Fresh	0.7	Feb-Apr (Winter to Spring)
*Carallumatuberculata*	Fresh	0.87	Feb-Apr (Winter to Spring)
*Carthamusoxyacantha*	Fresh	0.22	Jan-Mar (Winter)
*Daucuscarota*	Fresh	0.43	Dec-Mar (Winter)
*Diospyruslotus*	Dried	0.87	Throughout winter
*Dryopterisjuxtapostia*	Fresh	0.39	Apr-Aug (Summer)
*Dryopterisodontoloma*	Fresh	0.35	Apr-Aug (Summer)
*Ficus palmata*	Fresh and dried	0.43	Jul-Aug (Summer
*Malvasylvestris*	Fresh	0.95	Dec-March (Winter)
*Mentharoyleana*	Fresh and dried	0.087 per bundle	Mar-Aug (Summer
*Monothecabuxifolia*	Fresh	1.74	May-Jul (Summer)
*Morus alba*	Fresh	0.22	May-Jul (Summer)
*Moruslaevigata*	Fresh and dried	0.22	May-Jul (Summer)
*Nasturtium officinale*	Fresh	0.3	April-June (Summer)
*Portulacaoleracea*	Fresh	1.09	Jun-Aug (Summer)
*Trifoliumrepens*	Fresh	0.4	Dec-March (Winter)
*Vitisvinifera*	Fresh and dried	1.5	May-Jul (Summer)
*Ziziphus jujube*	Fresh and dried	0.43	Jul-Sep (Summer)
*Zizyphusmauritiana*	Fresh and dried	0.22	Jul-Sep (Summer)

### Biocultural and ecological interpretation of Jaccard similarity patterns and novelty

The highest Jaccard similarity values were recorded between Swat District and neighboring mountain regions, especially Kohistan (JI = 0.151; 32 shared species) [77], Upper Dir (JI = 0.136; 28 shared species) [76], and Neelum Valley (JI = 0.140; 30 shared species) [36]. Although these values are moderate in absolute terms, they represent the highest ethnobotanical similarities observed among all comparative regions in Pakistan, indicating that wild edible plant use in Swat is most closely aligned with ecologically contiguous mountain areas.Ecologically, all four regions share comparable altitudinal ranges (approximately 800–3,000 m a.s.l.), climatic conditions, and vegetation types, including temperate coniferous forests, montane shrublands, and alpine pastures. These environments support similar pools of wild edible plant taxa, particularly perennial herbs and shrubs adapted to cool temperate conditions. The relatively high number of shared species (28 to 32 taxa) suggests that ecological availability plays a major role in structuring wild food plant selection across these mountain regions.Culturally, Swat, Kohistan, Upper Dir, and Neelum Valley are characterized by agro-pastoral subsistence systems that integrate rain-fed agriculture, seasonal livestock grazing, and the use of forest and rangeland resources. Seasonal transhumance, shared grazing areas, and long-standing social connections among mountain communities facilitate the transmission and maintenance of traditional ecological knowledge. The convergence of relatively high JI values (0.136–0.151) with a high number of shared taxa therefore reflects the combined influence of ecological continuity and culturally embedded subsistence practices, demonstrating strong biocultural affinities between Swat and adjacent mountain regions (Table 5).

In contrast, markedly lower Jaccard similarity values were observed between Swat District and lowland regions, specifically the semi-arid region of Punjab, the peri-urban area of north-western Punjab, and Jhelum District. Comparisons with studies from the semi-arid region of Punjab showed very low similarity (JI = 0.044; 8 shared species) [83], while studies from the peri-urban area of north-western Punjab reported similarly limited overlap (JI = 0.054; 12 shared species) [70]. Comparison with Jhelum District also yielded minimal similarity (JI = 0.057; 13 shared species) [82]. These values represent the lowest levels of ethnobotanical overlap detected in the analysis.These strong dissimilarities reflect pronounced ecological and cultural differences between mountain and lowland areas. Ecologically, the semi-arid and peri-urban regions of Punjab and Jhelum District are dominated by plains, lower elevations, warmer climates, and subtropical or highly modified agro-ecosystems. Intensive and irrigated agriculture has replaced much of the natural vegetation, severely limiting the presence of mountain-adapted wild edible plant species. The very small number of shared taxa (8–13 species) quantitatively reflects this ecological discontinuity. Cultural and socioeconomic factors further amplify these differences. In the semi-arid and peri-urban regions of Punjab, food systems are strongly market-oriented, with greater reliance on cultivated crops and purchased foods. Urban influence, wage labor, and agricultural intensification have reduced dependence on wild edible plants, leading to the erosion or marginalization of related traditional knowledge. Similar processes are evident in Jhelum District, where improved infrastructure and market access have diminished the role of wild food resources in daily diets. The consistently low JI values (0.044–0.057) therefore reflect both ecological constraints and cultural shifts away from wild plant use.

Species-level overlap analysis further highlights the distinctiveness of the Swat ethnobotanical assemblage. Of the 175 documented wild edible plant taxa, 63 species (36%) were completely novel, exceeding the expected number under a uniform distribution (E = 43.75; χ² contribution = 8.47). An additional 56 species (32%) showed low overlap, being reported in only 1–5 previous studies (E = 43.75; χ² = 3.43). Medium-overlap species (6–10 reports) accounted for 27 taxa (E = 43.75; χ² = 6.41), while high-overlap species (11–15 reports) comprised 29 taxa (E = 43.75; χ² = 4.97) as shown in (table 6). The overall chi-square value (χ² = 23.28, df = 3, *p* < 0.001) indicates a significant deviation from random expectations, with novel and weakly shared species occurring more frequently than expected by chance.

Taken together, these results demonstrate that wild edible plant knowledge in Swat District is highly localized and context-dependent, shaped by specific mountain environments and culturally embedded agro-pastoral subsistence strategies. The strong contrast between the highest similarity values (JI = 0.136–0.151; 28–32 shared species) and the lowest values (JI = 0.044–0.057; 8–13 shared species) provides robust evidence that the Swat dataset represents a distinct ethnobotanical system rather than a redundant subset of regional knowledge, underscoring Swat’s importance as a bioculturally significant mountain region.

**Table 5:** Comparison of wild edible plant assemblages using Jaccard similarity index (JI)

Study area	Reference	Species in previous studies(A)	Species in present study (B)	Common species (C)	Unique species (A + B − 2 C)	Jaccard index (JI)
Swat	[74]	61	175	32	172	0.157
Swat	[75]	48	175	20	183	0.099
Upper Dir	[76]	59	175	28	178	0.136
Kohistan	[77]	69	175	32	180	0.151
Chitral	[19]	44	175	19	181	0.095
Neelum Valley	[36]	70	175	30	185	0.140
Kurram	[35]	59	175	25	184	0.120
Waziristan	[79]	52	175	21	185	0.102
Hindu Kush	[78]	64	175	27	185	0.127
Gadoon Valley	[80]	51	175	21	184	0.102
Punjab	[81]	60	175	15	205	0.068
Punjab	[70]	60	175	12	211	0.054
Jhelum	[82]	64	175	13	213	0.057
Punjab	[83]	15	175	8	174	0.044
Balochistan	[84]	131	175	16	274	0.055
Balochistan	[83]	39	175	13	188	0.065
Balochistan	[84]	40	175	17	181	0.086

**Table 6:** Distribution of novel and overlapping plant species

Overlap range	O	E	(O-E)²	(O-E)²/E
0 (Novel)	63	43.75	370.5625	8.47
1–5	56	43.75	150.0625	3.43
6–10	27	43.75	280.5625	6.41
11–15	29	43.75	217.5625	4.97

**Table 7:** Ethnobotanical inventory of wild edible plants (WEPs) in Swat, Pakistan

Botanical name	Family	1. Voucher code	Local name	Habit	Used part	Gathering period	Traditional food use	FC	UR	UV	RFC
*Abies pindrow* (Royle ex D. Don) Royle	Pinaceae	2. SM001	Char	Tree	Leaves	Ever green	Used as substitute for tea	80	90	0.563	0.5
*Alopecurus pratensis* L.	Poaceae	3. SM002	Kopra Boty	Herb	Young inflorescence	Spring	Young inflorescence eaten like powdered coconut (mostly children)	40	45	0.281	0.25
*Amaranthus graecizans* L.	Amaranthaceae	4. SM003	Ghana	Herb	Leaves	Summer	Cooked as as vegetable (ghee, Garlic + tomato and red powder chilly)	70	75	0.469	0.44
*Amaranthus spinosus* L.	Amaranthaceae	5. SM004	Ganrhar	Herb	Young shoot	Summer	Cooked as as vegetable (ghee, Garlic + tomato and red powder chilly)	65	70	0.438	0.41
*Amaranthus viridis* L.	Amaranthaceae	6. SM005	Chalway	Herb	Young shoot	Summer	Cooked as as vegetable (ghee, Garlic + tomato and red powder chilly)	60	65	0.406	0.38
*Asparagus adscendens* Roxb	Asparagaceae	7. SM006	Tendonra	Herb	Young shoot	Summer	Cooked with egg or meat, also boiled in milk for better test	50	55	0.344	0.31
*Asparagus officinalis* L.	Asparagaceae	8. SM007	Tendonra	Herb	Young shoot	Summer	Cooked as as vegetable (ghee and Masala)	55	60	0.375	0.34
*Asparagus* spp.	Asparagaceae	9. SM008	Tendonra	Herb	Young shoot	Summer	Cooked with egg or meat or boiled in milk for pleasant test	45	50	0.313	0.28
*Asphodelus tenuifolius* Cavan	Asphodelaceae	10. SM009	Ogakay	Herb	Young shoot	Summer	It is eaten with maize bread or crushed and mixed into maize bread, then oil is added	35	40	0.25	0.22
*Asplenium ceterach* L.	Aspleniaceae	11. SM0010	Gawanjay	Herb	Young shoot	Spring	Cooked as vegetable	40	45	0.281	0.25
*Astragalus anisacanthus* Boiss.	Fabaceae	12. SM0011	Mamol	Shrub	Young shoot	Summer	Cooked as vegetable	25	30	0.188	0.16
*Bauhinia variegata* L.	Fabaceae	13. SM0012	Kulyer	Tree	Bark and Flower heads	Summer	Cooked as vegetable	50	55	0.344	0.31
*Berberis lycium* Royle	Berberidaceae	14. SM0013	Kwary	Shrub	Fruits	Summer	Fresh fruits are taken to quench thirst or for testing	120	130	0.813	0.75
*Berberis vulgaris* L.	Berberidaceae	15. SM0014	Kwaray	Shrub	Fruits	Summer	Fresh fruits are taken to quench thirst or for testing	100	105	0.656	0.63
*Brassica juncea* (L.) Czern.	Brassicaceae	16. SM0015	Jawawa	Herb	Young shoot	Winter	Used as salad	55	60	0.375	0.34
*Brassica rapa* L.	Brassicaceae	17. SM0016	Sharsham	Herb	Seed	Winter to Spring	Leaves cooked as vegetable and seed oil used for cooking	45	50	0.313	0.28
*Caltha alba* Camb.	Ranunculaceae	18. SM0017	Makhan Path	Herb	Young shoot	Spring	Cooked as a vegetable: boiled, drained, and then sauteed with garlic, tomato, and ghee	20	25	0.156	0.13
*Capsella bursa-pastoris* (L.) Medic.	Brassicaceae	19. SM0018	Bambiasa	Herb	Young shoot	Spring	Cooked as vegetable	40	45	0.281	0.25
*Prunella vulgaris* L	Lamiaceae	20. SM0019	Kali	Herb	Young shoot	Winter	Cooked as vegetable	50	55	0.344	0.31
*Caralluma tuberculata* N.E. Brown	Asclepiadaceae	21. SM0020	Pamankay	Herb	Young shoot	Winter	Cooked as vegetable	45	50	0.313	0.28
*Cardamine hirsuta* L.	Brassicaceae	22. SM0021	Tarmera	Herb	Young shoot	Winter to Summer	Cooked as a vegetable: boiled in water, then sautéed with garlic, tomato, ghee, and chili powder; also used raw in salads	35	40	0.25	0.22
*Carthamus oxyacantha* M. Bieb	Asteraceae	23. SM0022	Kaeeza	Herb	Young shoot	Spring	Locally, it is crushed along with other plants such as garlic, coriander, Allium, and others, then boiled, and rice is added to make a special dish locally called ‘Chukan	30	35	0.219	0.19
*Carum carvi* L.	Lamiaceae	24. SM0023	Toory Zanki	Herb	Seed	Summer	Seeds used as spice	25	30	0.188	0.16
*Carum copticum*L.	Apiaceae	25. SM0024	Zankay	Herb	Seed	Summer	Seeds used as spice for cooking specialyespecially in Karai (meat)	35	40	0.25	0.22
*Celosia argentea* L.	Amaranthaceae	26. SM0025	Charkhowal	Herb	Leaves	Spring	Cooked as vegetable	25	30	0.188	0.16
*Celtis australis* L.	Ulmaceae	27. SM0026	Tagha	Tree	Fruits	Summer	Taken as fruit	40	45	0.281	0.25
*Celtis caucasica* L.	Ulmaceae	28. SM0027	Tagha	Tree	Fruits	Summer	Taken as fruit	35	40	0.25	0.22
*Chenopodium album* L.	Amaranthaceae	29. SM0028	Sarmay	Herb	Young shoot	Summer	Cooked as a vegetable: boiled in water, then sautéed with garlic, tomato, ghee, and chili powder;	100	110	0.688	0.63
*Chenopodium murale* L.	Amaranthaceae	30. SM0029	Chalwairay	Herb	Young shoot	Summer	Cooked as a vegetable: boiled in water, then sautéed with garlic, tomato, ghee, and chili powder;	60	65	0.406	0.38
*Cichorium intybus* L.	Asteraceae	31. SM0030	Haan	Herb	Young shoot	Summer	Cooked as a vegetable: boiled in water, then sautéed with garlic, tomato, ghee, and chili powder;	55	60	0.375	0.34
*Coccinia grandis* (L.) Voigt	Cucurbitaceae	32. SM0031	Kanduri	Herb	Young shoot	Any Season	Cooked as vegetable	50	55	0.344	0.31
*Cotoneaster affinis* Lindl.	Rosaceae	33. SM0032	Mamanara	Shrub	Fruits	Summer	Taken as fruit	30	35	0.219	0.19
*Cotoneaster microphyllus* Wall. ex Lindley	Rosaceae	34. SM0033	Mamanara	Shrub	Fruits	Summer	Taken as fruit	20	25	0.156	0.13
*Cotoneaster nummularia* Fisch & Mey	Rosaceae	35. SM0034	Kharawa	Shrub	Fruits	Summer	Taken as fruit	40	45	0.281	0.25
*Crataegus oxyacantha* L.	Rosaceae	36. SM0035	Changa	Tree	Fruits	Summer	Taken as fruit	35	40	0.25	0.22
*Cuminum cyminum* L.	BrassicaceaeApiaceae	37. SM0036	Zeera	Herb	Seed	Summer	Used as spice and flavoring agent	30	35	0.219	0.19
*Cupressus torulosa* D. Don	Cupressaceae	38. SM0037	Wara Serwa	Tree	Seed	Summer	Oil obtained from seed	25	30	0.188	0.16
*Cymbopogon citratus* Springs	Poaceae	39. SM0038	Sheen Chay	Herb	Young shoot	Summer	Used to flavor tea and particularly appreciated locally for traditional occasions such as marriages and other ceremonies	20	25	0.156	0.13
*Daphne oloides* Schreb.	Thymellaeaceae	40. SM0039	Laighionai	Shrub	Fruits	Summer	Taken as fruit	50	55	0.344	0.31
*Daucus carota* L.	Apiaceae	41. SM0040	Gazara	Herb	Young shoot	Winter	The leaves are cooked as a vegetable, while the tubers are eaten raw.	45	50	0.313	0.28
*Debregeasia salicifolia* (D. Don) Rendle	Urticaceae	42. SM0041	Ajlai	Tree	Fruits	Summer	Taken as fruit	35	40	0.25	0.22
*Descurainia sophia* (L.) Webb ex Prantl	Brassicaceae	43. SM0042	Jenjar	Herb	Leaves	Summer	Cooked as as vegetable	30	35	0.219	0.19
*Digera muricata* (L.) Mart.	Amaranthaceae	44. SM0043	Sur Gualy	Herb	Leaves	Summer	Cooked as a vegetable: boiled in water, then sautéed with garlic, tomato, ghee, and chili powder;	25	30	0.188	0.16
*Diospyrus lotus* L.	Ebenaceae	45. SM0044	Toor amlook	Tree	Fruits	Summer	Taken as fruit	20	25	0.156	0.13
*Duchesnea indica* (Andr) Folke	Rosaceae	46. SM0045	Zmaki Toth	Herb	Fruits	Spring & Summer	Taken as fruit	35	40	0.25	0.22
*Elaeagnus parviflora* Wall. ex Royle	Elaeagnaceae	47. SM0046	Ghanam Ragay	Tree	Fruits	Summer	Taken as fruit	30	35	0.219	0.19
*Elaeagnus umbellata* Thumb.	Elaeagnaceae	48. SM0047	Ghanam Ranga	Shrub	Fruits	Summer	Taken as fruit	20	25	0.156	0.13
*Elettaria cardamomum* (L.) Maton	Zingiberaceae	49. SM0048	Lachii	Shrub	Whole plant	Summer	Known for its aroma, it is used as a cooking spice and to flavor tea	25	30	0.188	0.16
*Eruca sativa* Mill	Brassicaceae	50. SM0049	Javava	Herb	Fruits	Summer	Taken as fruit	20	25	0.156	0.13
*Eryngium caeruleum* M. Bieb.	Apiaceae	51. SM0050	Pesho Panja	Herb	Young shoot	Summer	Eaten raw, especially by children	15	20	0.125	0.09
*Euphorbia prostrata* Ait.	Euphorbiaceae	52. SM0051	Ghozakay	Herb	Flower	Summer	Eaten raw, especially by children	50	55	0.344	0.31
*Ficus carica* Forsk.	Moraceae	53. SM0052	Inzar	Tree	Fruits	Summer	Taken as fruit	45	50	0.313	0.28
*Ficus palmata* Forsk.	Moraceae	54. SM0053	Inzar	Tree	Fruits	Summer	Taken as fruit	35	40	0.25	0.22
*Foeniculum vulgare* Mill.	Apiaceae	55. SM0054	Kagynalay	Herb	Seed & Young shoot	Summer	Used as a flavoring agent	30	35	0.219	0.19
*Fragaria indica* Andrew	Rosaceae	56. SM0055	Zmaki Toth	Herb	Fruits	Spring & Summer	Taken as fruit	25	30	0.188	0.16
*Fragaria nubicola* (Lindl. ex Hook. f.) Lacaita	Rosaceae	57. SM0056	Zmaki Toth	Herb	Fruits	Summer	Taken as fruit	20	25	0.156	0.13
*Grewia asiatica* L.	Malvaceae	58. SM0057	Pastoni	Tree	Fruits	Summer	Taken as fruit	35	40	0.25	0.22
*Helianthus annuus* L.	Asteraceae	59. SM0058	Namarparas	Herb	Seed	Summer	The seed oil is used for cooking, and the seeds are also cooked with salt and eaten, mostly by children and young people	25	30	0.188	0.16
*Heliotropium ovalifolium* Forssk.	Boraginaceae	60. SM0059	Sharay	Herb	Young shoot	Spring	Cooked as vegetable	40	45	0.281	0.25
*Hippophae rhamnoides* L.	Elaeagnaceae	61. SM0060	Sabak Than	Tree	Fruits	Summer	Taken as fruit	20	25	0.156	0.13
*Indigofera* sp.	Fabaceae	62. SM0061	Gedarghowag	Shrub	Young shoot	Early Summer	Flowers and leaves eaten raw food	25	30	0.188	0.16
*Lactuca abietina* (Boiss.&Balansa) Bornm.	Asteraceae	63. SM0062	Kao	Herb	Young shoot	Spring	Used as salad and eaten raw	30	35	0.219	0.19
*Lathyrus aphaca* L.	Fabaceae	64. SM0063	Chelo Paliy	Herb	Pods	Summer	Eaten raw by children for its taste	28	32	0.2	0.18
*Lathyrus cicera* L.	Fabaceae	65. SM0064	Chilo	Herb	Young shoot & Pod	Spring	Pods are eaten raw, while the shoots are cooked as a vegetable	32	37	0.231	0.2
*Lathyrus sativus* L.	Fabaceae	66. SM0065	Ghata Chilo	Herb	Young shoot	Spring	Mixed with saags and cooked as vegetable	35	40	0.25	0.22
*Lepidium rudirale* HK & Anders non L.	Brassicaceae	67. SM0066	Zangali Alam	Herb	Leaves	Summer	Leaves used as salad	25	30	0.188	0.16
*Lepidium sativum* L.	Brassicaceae	68. SM0067	Alaam	Herb	Young shoot	Winter to Spring	Mixed with saags and cooked as vegetable	30	35	0.219	0.19
*Malva neglecta* Wall.	Malvaceae	69. SM0068	Panirak	Herb	Young shoot	Winter	Boiled, drained, and then sautéed with ghee, garlic, and tomato; eaten as a vegetable	15	18	0.113	0.09
*Malva sylvestris* L.	Malvaceae	70. SM0069	Sanchal	Herb	Young shoot or Leaves	Early winter	Boiled, drained, and then sautéed with ghee, garlic, and tomato; eaten as a vegetable	18	20	0.125	0.11
*Marsilea minuta* L.	Marsileaceae	71. SM0070	Chopatra	Herb	Young shoot	Summer	Boiled, drained, and then sautéed with ghee, garlic, and tomato; eaten as a vegetable	25	30	0.188	0.16
*Marsilea quadrifolia* L.	Marsileaceae	72. SM0071	Chopatra	Herb	Leaves	Summer	Boiled, drained, and then sautéed with ghee, garlic, and tomato; eaten as a vegetable	30	35	0.219	0.19
*Medicago polymorpha* L.	Fabaceae	73. SM0072	Shpeshtra	Herb	Young shoot	Spring to Summer	Boiled, drained, and then sautéed with ghee, garlic, red chilli and tomato; eaten as a vegetable	28	33	0.206	0.18
*Mentha arvensis* L.	Lamiaceae	74. SM0073	Podina	Herb	Young shoot	Summer	Used as flavoring agent and also used for sauce	25	30	0.188	0.16
*Mentha longifolia* (L.) Huds.	Lamiaceae	75. SM0074	Velanay	Herb	Young shoot	Summer or Spring	Cooked with maize cobs as a flavoring agent and mixed with other leafy vegetables for the best taste and aroma	20	25	0.156	0.13
*Mentha royleana* Wall. ex Benth.	Lamiaceae	76. SM0075	Podina	Herb	Young shoot	Spring to Winter	Used as flavoring agent and also used for sauce	22	26	0.163	0.14
*Mirabilis jalapa* L.	Nyctaginaceae	77. SM0076	Guli Badi	Herb	Leaves	Summer	Used as a food coloring or as an ingredient in food	30	35	0.219	0.19
*Momordica dioica* Roxb. ex Willd	Cucurbitaceae	78. SM0077	Kakora	Herb	Fruits	Winter	Cooked as a vegetable, similar to bitter gourd	50	55	0.344	0.31
*Monotheca sideroxylon* (Falc) A. DC.	Sapotaceae	79. SM0078	Gowargora	Shrub	Fruits	Summer	Taken as fruit	35	40	0.25	0.22
*Morus alba* L.	Moraceae	80. SM0079	Baidana	Tree	Fruits	Summer	Taken as fruit	45	50	0.313	0.28
*Morus lavaegata* Wallich ex Brandis	Moraceae	81. SM0080	Shahtooth	Tree	Fruits	Summer	Taken as fruit	25	30	0.188	0.16
*Morus nigra* L.	Moraceae	82. SM0081	Toth	Tree	Fruits	Summer	Taken as fruit	18	22	0.138	0.11
*Myrtus communis* L.	Myrtaceae	83. SM0082	Manroo	Shrub	Fruits	Summer	Taken as fruit	15	18	0.113	0.09
*Mysine africana* L.	Myrsinaceae	84. SM0083	Manrgawaya	Herb	Fruits	Summer	Taken as fruit	40	45	0.281	0.25
*Nannorrhops ritchieana* (Griff.) –Aitch	Arecaceae	85. SM0084	Mianzarai	Shrub	Fruits	Early winter	Taken as fruit	20	25	0.156	0.13
*Nasturtium officinale* R. Br.	Brassicaceae	86. SM0085	Tarmeera	Herb	Young shoot	Winter, Spring & Summer	Boiled, drained, and then sautéed with ghee, garlic, and tomato; eaten as a vegetable	35	40	0.25	0.22
*Oenothera rosea* Soland.	Onagraceae	87. SM0086	Sordingy	Herb	Young shoot	Summer	Used to prepare herbal tea.	30	35	0.219	0.19
*Olea europaea* L.	Oleaceae	88. SM0087	Khona	Tree	Fruits	Summer	The oil is used in cooking and can also be consumed raw	25	30	0.188	0.16
*Olea ferruginea* Royle	Oleaceae	89. SM0088	Khona	Tree	Fruits	Summer	The oil is used in cooking and can also be consumed raw	18	22	0.138	0.11
*Onopordum acanthium* L.	Asteraceae	90. SM0089	Warejakay	Herb	Seed	Summer	The seeds are eaten raw, especially by children	35	40	0.25	0.22
*Onosma hispida* Wall.ex G.Don	Boraginaceae	91. SM0090	Abaiy Pay	Herb	Flower	Summer	Children suck the flowers for their sweet taste.	30	35	0.219	0.19
*Opuntia dillenii* (Ker Gawl.) Haw.	Cactaceae	92. SM0091	Tafnra	Shrub	Fruits	Summer	Taken as fruit	18	22	0.138	0.11
*Oxalis corniculata* L.	Oxalidaceae	93. SM0092	Mangazay	Herb	Young shoot	Spring	Young shoots used as salad	20	25	0.156	0.13
*Oxalis stricta* L.	Oxalidaceae	94. SM0093	Tarookay	Herb	Young shoot	Summer	cooked as vegetable and used as salad	22	26	0.163	0.14
*Papaver pavoninum* Schrenk	Papaveraceae	95. SM0094	Riday	Herb	Young shoot	Spring	Cooked as vegetable and also mixed with other saag	28	33	0.206	0.18
*Papaver rhoeas* L.	Papaveraceae	96. SM0095	Redaay	Herb	Leaves	Spring	Cooked as vegetable and also mixed with other saag	25	30	0.188	0.16
*Persicaria plebejum* R. Br	Polygonaceae	97. SM0096	Bandakay	Herb	Young shoot	Summer	Cooked as vegetable	30	35	0.219	0.19
*Physalis minima* L	Solanaceae	98. SM0097	Tamater	Herb	Fruits & Young shoot	Summer	Fruits are either cooked as a vegetable or eaten raw.	22	26	0.163	0.14
*Pinus gerardiana* Wall. ex Lamb	Pinaceae	99. SM098	Changhozi	Tree	Seed	Winter	Seeds eaten raw and used as dry fruit	28	33	0.206	0.18
*Plantago lanceolatum* L.	Plantaginaceae	100. SM099	Jabbay	Herb	Young shoot	Spring & Summer	Cooked as vegetable and also mixed with other saag for better test	30	35	0.219	0.19
*Plantago major* L.	Plantaginaceae	101. SM0100	Ispaghol	Herb	Leaves	Summer	Cooked as vegetable and also mixed with other saag for better test	18	22	0.138	0.11
*Plantago ovate ovata*Forssk.	Plantaginaceae	102. SM0101	Speghol	Tree	Seed	Summer	Edible seeds	28	33	0.206	0.18
*Polygonum aviculare* L.	Polygonaceae	103. SM0102	Bandakay	Herb	Young shoot	Spring to Winter	Cooked as vegetable	35	40	0.25	0.22
*Polygonum plebejum* Boiss	Polygonaceae	104. SM0103	Bandakay	Herb	Young shoot	Summer to Winter	Cooked as vegetable	30	35	0.219	0.19
*Polystichum lonchitis* (L.) Roth	Dryopteridaceae	105. SM0104	Gawanjay	Herb	Young shoot	Spring	Cooked as vegetable	18	22	0.138	0.11
*Portulaca oleracea* L.	Portulaceae	106. SM0105	Warkharay	Herb	Fruits	Summer	Boiled, drained, and then sautéed with ghee, garlic, and tomato; can also be cooked with yogurt	20	25	0.156	0.13
*Portulaca quadrifida* L.	Portulaceae	107. SM0106	Warkharay	Herb	Young shoot	Summer	Boiled, drained, and then sautéed with ghee, garlic, and tomato; can also be cooked with yogurt	35	40	0.25	0.22
*Portulaca tuberosa* Roxb.	Portulaceae	108. SM0107	Warkharay	Herb	Young shoot	Summer	Boiled, drained, and then sautéed with ghee, garlic, and tomato; can also be cooked with yogurt	40	45	0.281	0.25
*Poterium polygonum* Waldst & Kit.	Rosaceae	109. SM0108	–	Herb	Leaves	Summer	Leaves used as salad and eaten as fresh	25	30	0.188	0.16
*Pteridium aquilinum* (L.) Kuhn	Dennstaedtiaceae	110. SM009	Gownjay	Herb	Young shoot	After winter	Cooked with yogurt and eaten with maize bread	30	35	0.219	0.19
*Punica granatum* L.	Punicaceae	111. SM0110	Nagoray	Tree	Fruits	Summer	Taken as fruit	40	45	0.281	0.25
*Pyrus pashia* Buch-Ham ex D. Don	Rosaceae	112. SM0111	Gedar Tanga	Tree	Fruits	Winter	Taken as fruit	45	50	0.313	0.28
*Pyrus* spp.	Rosaceae	113. SM0112	Tangay	Tree	Fruits	Summer	Taken as fruit	35	40	0.25	0.22
*Quercus baloot* Griff.	Fagaceae	114. SM0113	Banj	Tree	Fruits	Winter	Consumed as a fruit: locally, it is either salted and heated over fire for some time or directly placed on fire to reduce bitterness before eating	25	30	0.188	0.16
*Quercus dilatata* Lindl ex Royle	Fagaceae	115. SM0114	Banj	Tree	Fruits	Winter	Consumed as a fruit: locally, it is either salted and heated over fire for some time or directly placed on fire to reduce bitterness before eating	28	33	0.206	0.18
*Quercus incana* Roxb.	Fagaceae	116. SM0115	Toor Banj	Tree	Fruits	Winter	Consumed as a fruit: locally, it is either salted and heated over fire for some time or directly placed on fire to reduce bitterness before eating	20	25	0.156	0.13
*Quercus semecarpifolia* Sm.	Fagaceae	117. SM0116	–	Tree	Fruits	Winter	Consumed as a fruit: locally, it is either salted and heated over fire for some time or directly placed on fire to reduce bitterness before eating	30	35	0.219	0.19
*Rhus chinensis* Miller	Anacardiaceae	118. SM0117	Titray	Shrub	Fruits	Summer	Eaten as raw food	35	40	0.25	0.22
*Rosa canina* L.	Rosaceae	119. SM0118	Gharaiz Gul	Climber	Fruits	Winter	Taken as fruit	28	33	0.206	0.18
*Rosa moschata* Herrm	Rosaceae	120. SM0119	Khwrach	Shrub	Fruits	Summer to Winter	Taken as fruit	30	35	0.219	0.19
*Rosa webbiana* Wallich ex Royle	Rosaceae	121. SM0120	Zangali Gulab	Climber	Fruits	Winter	Taken as fruit	25	30	0.188	0.16
*Rubia cordifolia* L	Rubiaceae	122. SM0121	Kargha Posky	Herb	Fruits	Winter	Taken as fruit	18	22	0.138	0.11
*Rubus biflorus* Ham. ex Sm.	Rosaceae	123. SM0122	Ach	Shrub	Fruits	Summer	Taken as fruit	28	33	0.206	0.18
*Rubus ellipticus* Smith	Rosaceae	124. SM0123	Gooraj	Herb	Fruits	Summer	Taken as fruit	25	30	0.188	0.16
*Rubus fruticosus* G.N. Jones	Rosaceae	125. SM0124	Karwara	Shrub	Fruits	Summer	Taken as fruit	30	35	0.219	0.19
*Rubus niveus* Thumb. non Wall.	Rosaceae	126. SM0125	Baganra	Shrub	Fruits	Summer	Taken as fruit	28	33	0.206	0.18
*Rubus sanctus* Schreb.	Rosaceae	127. SM0126	Zmaki Toth	Herb	Fruits	Summer	Taken as fruit	25	30	0.188	0.16
*Rubus ulmifolius* Schott	Rosaceae	128. SM0127	Karwara	Shrub	Fruits	Summer	Taken as fruit	22	26	0.163	0.14
*Rumex alpinuse*L	Scrophulariaceae	129. SM0128	Da ghra shulkhay	Herb	Young shoot	Spring	Cooked as vegetable like spinchspinach	18	22	0.138	0.11
*Rumex dentatus* L.	Polygonaceae	130. SM0129	Shalkhay	Herb	Leaves	Spring to Winter	Cooked as Vegetable or mixed with other saag	20	25	0.156	0.13
*Rumex hastatus* D. Don	Polygonaceae	131. SM0130	Tarookay	Herb	Leaves	Spring to Summer	Mixed with other saag for its test or used in chutney	30	35	0.219	0.19
*Rumex nepalensis* Spreng.	Polygonaceae	132. SM0131	Ovowal	Herb	Leaves	Winter to Spring	Cooked as Vegetable or mixed with other saag	25	30	0.188	0.16
*Sageretia brandrethiana* Aitch.	Rhamnaceae	133. SM0132	Mamanara	Shrub	Fruits	Summer	Taken as fruit	18	22	0.138	0.11
*Sageretia thea* (Osbeck) M.C. Johnst	Rhamnaceae	134. SM0133	Momnara	Shrub	Fruits	Summer	Taken as fruit	20	25	0.156	0.13
*Salvia lanata* Roxb.	Lamiaceae	135. SM0134	Mattar Jarrai	Herb	Young shoot	Summer	Cooked as Vegetable	25	30	0.188	0.16
*Salvia moorcroftiana* Wall. ex Benth.	Lamiaceae	136. SM0135	Khar Ghwag	Herb	Young shoot	Spring	Young stems eaten as raw food mostly children	22	26	0.163	0.14
*Scandix pecten-veneris* L.	Apiaceae	137. SM0136	Gangahay	Herb	Young shoot	Spring	Eaten raw or mixed with other saag	25	30	0.188	0.16
*Sideroxylon mascatense* (A.DC.) T.D. Penn	Sapotaceae	138. SM0137	Gwargwara	Tree	Fruits	Summer	Taken as fruit	30	35	0.219	0.19
*Silene conidia* L.	Caryophylaceae	139. SM0138	Mangotay	Herb	Young shoot	Spring	Cooked with saag as Vegetable	28	33	0.23	0.16
*Silybum marianum* (L.) Gaertn.	Asteraceae	140. SM0139	Warejakay	Herb	Seed	Summer	Seeds eaten raw mostly children	30	35	0.219	0.19
*Sisymbrium altissimum* L.	Brassicaceae	141. SM0140	Awaray	Herb	Young shoot	Winter to Spring	Cooked as Vegetable	25	30	0.188	0.16
*Sisymbrium irio* L.	Brassicaceae	142. SM0141	Awaray	Herb	Leaves	Spring to Summer	Cooked as Vegetable	28	32	0.2	0.18
*Sisymbrium officinale* L.	Brassicaceae	143. SM0142	Shersham	Herb	Leaves	Winter	Cooked as Vegetable or taken as salad	25	30	0.188	0.16
*Solanum melongena* L.	Solanaceae	144. SM0143	Kachmachoo	Herb	Leaves & Fruits	Spring to Winter	Fruits eaten raw and leaves cooked as vegetable	28	33	0.206	0.18
*Solanum miniatum* Benth. ex Willd.	Solanaceae	145. SM0144	Kachmachoo	Herb	Young shoot	Summer	Vegetable	25	30	0.188	0.16
*Solanum nigrum* L.	Solanaceae	146. SM0145	Kachmachoo	Herb	Young shoot	Summer	Fruits eaten raw and leaves cooked as vegetable	30	35	0.219	0.19
*Solanum villosum* Mill.	Solanaceae	147. SM0146	Kachmachoo	Herb	Leaves & Fruits	Spring to Winter	Fruits eaten raw, leaves cooked as vegetable	28	33	0.206	0.18
*Stellaria media* (L.) Vill.	Caryophylaceae	148. SM0147	Olalai	Herb	Young shoot	Spring	Cooked Vegetable along with *Trifolium* spp	25	30	0.188	0.16
*Syzygium aromaticum* (L.) Merr. Perry	Myrtaceae	149. SM0148	–	Tree	Fruits & Flower	Summer	Fruits and flowers used in curries	20	25	0.156	0.13
*Thymus linearis* Benth.	Lamiaceae	150. SM0159	–	Herb	Young shoot	Summer to Winter	Aerial parts used in spices	25	30	0.188	0.16
*Thymus serphyllum* L.	Lamiaceae	151. SM0150	Da Ghra Speraky	Herb	Young shoot	Summer	Used to prepare herbal tea	20	25	0.156	0.13
*Trianthema portulacastrum* L.	Aizoaceae	152. SM0151	Ghanay	Herb	Young shoot	Summer	Cooked as Vegetable	28	33	0.206	0.18
*Trifolium repens* L.	Fabaceae	153. SM0152	Showtal	Herb	Young shoot	Winter	Cooked as Vegetable: very famous saag in the area	25	30	0.188	0.16
*Urtica dioica* L.	Urticaceae	154. SM0150	Seezonkay	Herb	Young shoot	Summer	Cooked as vegetable	30	35	0.219	0.19
*Urtica urens* L.	Urticaceae	155. SM0154	Sezonki	Herb	Young shoot	Spring	Boiled, drained, and then sautéed with ghee, garlic, and tomato; eaten as a vegetable	20	25	0.156	0.13
*Veronica anagallis aquatica* L.	Scrophulariaceae	156. SM0150	Surkhay	Herb	Young shoot	Spring	Cooked as Vegetable or mixed cooked with *Trifolium*	25	30	0.188	0.16
*Viburnum cotinifolium* D. Don	Viburnaceae	157. SM0156	Kasar Botay	Shrub	Fruits	Summer	Taken as fruit	28	33	0.206	0.18
*Viburnum foetens* Decne	Viburnaceae	158. SM0157	Khapyanga	Shrub	Fruits	Summer	Taken as fruit	25	30	0.188	0.16
*Viburnum grandiflorum* Wall. ex DC.	Viburnaceae	159. SM0158	Ghaza	Shrub	Fruits	Summer	Taken as fruit	25	30	0.188	0.16
*Viburnum mullaha* Decne	Viburnaceae	160. SM0159	Khapyanga	Shrub	Fruits	Summer	Taken as fruit	28	33	0.206	0.18
*Viburnum nervosum* D. Don	Viburnaceae	161. SM0160	Asos	Shrub	Fruits	Summer	Taken as fruit	20	25	0.156	0.13
*Vicia hirsuta* (L.) Gray	Fabaceae	162. SM0161	Palay	Herb	Pods	Spring	Eaten raw or keep on fire along with salt for better test	25	30	0.188	0.16
*Vicia monantha* Retz.	Fabaceae	163. SM0162	Palay	Herb	Pods	Spring	Eaten raw or keep on fire along with salt for better test	28	33	0.206	0.18
*Vicia sativa* L.	Fabaceae	164. SM0163	Marghay Khapa	Herb	Fruits & Leaves	Spring	Eaten raw or keep on fire along with salt for better test, also leaves as vegetable	25	30	0.188	0.16
*Vicia tetrasperma* (L.) Schreb.	Fabaceae	165. SM0164	Palay	Herb	Pods	Spring	Eaten raw or keep on fire along with salt for better test	30	35	0.219	0.19
*Vigna aconitifolia* (Jacq.) Marechal	Fabaceae	166. SM0165	–	Climbing Herb	Leaves & Seed	Summer	Cooked as as vegetable and seeds eaten as raw food	25	30	0.188	0.16
*Vitis vinifera* L.	Vitaceae	167. SM0166	Kawar	Climber	Fruits	Summer	Taken as fruit	30	35	0.219	0.19
*Zanthoxylum alatum* DC.	Rutaceae	168. SM0167	Dambara	Tree	Fruits	Summer	Flavoring agent and mostly and widely used in sauce	28	33	0.206	0.18
*Zanthoxylum armatum* DC.	Rutaceae	169. SM0168	Dambara	Tree	Seed	Spring to Summer	Flavoring agent and mostly and widely used in sauce	25	30	0.188	0.16
*Ziziphus jujuba* Mill	Rhamnaceae	170. SM0169	Markhnray	Tree	Fruits	Summer	Taken as fruit	28	33	0.206	0.18
*Ziziphus nummularia* (Burm. f.) Wight & Arn.	Rhamnaceae	171. SM0170	Kurkanda	Shrub	Fruits	Summer	Taken as fruit	25	30	0.188	0.16
*Ziziphus oxyphylla* Edgew.	Rhamnaceae	172. SM0171	Enalay	Shrub	Fruits	Summer to Winter	Taken as fruit	20	25	0.156	0.13
*Ziziphus spina-christi* (L.) Desf.	Rhamnaceae	173. SM0172	Markhnray	Tree	Fruits	Summer	Taken as fruit	25	30	0.188	0.16
*Zizyphus mauritiana* Lam.	Rhamnaceae	174. SM0173	Mada Bera	Tree	Fruits	Winter	Taken as fruit	30	35	0.219	0.19
*Zizyphus sativa* Gaertn.	Rhamnaceae	175. SM0174	Markhnray	Tree	Fruits	Summer	Taken as fruit	25	30	0.188	0.16
*Zizyphus vulgaris* L.	Rhamnaceae	176. SM0175	Markhnray	Tree	Fruits	Summer	Taken as fruit	28	33	0.206	0.18

## Discussion

The present study represents a comprehensive ethnobotanical inventory of wild edible plants (WEPs) in Swat District, Pakistan, documenting 175 species across 72 families. This highlights the region’s rich biocultural heritage and the crucial role of WEPs in local diets, livelihoods, and traditions. Swat’s status as a biodiversity hotspot in the Hindu Kush-Himalayan region, with altitudes ranging from (600 to 5,957 m) and varied ecological zones fosters heterogeneous vegetation from temperate forests to alpine meadows [10, 64]. Herbaceous taxa dominated the recorded species reflecting preference for easily accessible fast-growing plants in both anthropogenic and natural environments. Although, young shoots, fruits, and leaves were most frequently utilized primarily as vegetables, fruits or raw snacks. Such patterns are consistent with both regional and global ethnobotanical trends where WEPs from disturbed or human-modified habitats including weedy species, are favored for their ecological resilience and availability during periods of food scarcity [18, 20, 50, 71].

Comparisons with Pakistani ethnobotanical studies confirm the uniqueness of Swat’s WEP assemblage. Lalku Valley, Swat [74], and Kumrat Valley, Upper Dir [75] share taxa such as *Berberis lycium* and *Mentha* spp., yet Swat shows greater species richness and novel modes of consumption, including raw uses. Kohistan Upper KP [76], North Waziristan [77] and Hindu Kush tribal areas [78] exhibit partial overlap with select species whereas Gadoon Valley [79], peri-urban Punjab [80, 81], and Balochistan [82–84] show minimal similarity. These distinctions supported by low Jaccard similarity indices (0.044–0.157) emphasize the influence of local ecology, altitude, land use and culturally-specific dietary practices on WEP selection and utilization.

### Cultural and quantitative significance of WEPs

Quantitative analyses underline the cultural and nutritional importance of key taxa. Berberis lycium displayed the highest use value (UV) and relative frequency of citation (RFC), followed by *Chenopodium album* and *Berberis vulgaris* indicating their integration into both daily diets and traditional beverages. The high indices for these species, often weeds or hardy shrubs, underscore their resilience and importance for food security in nutritionally vulnerable mountain communities, particularly in regions where national-level dietary insecurity is significant [5, 27].

Cooked vegetables such as *Nasturtium officinale*, *Trifolium repens* and *Amaranthus viridis* prepared as “taarka” or “chokaner,” mirror findings from northern Pakistan [3, 7] and are documented in Lalku Valley [74], Kumrat [75], and Gadoon Valley [79] reflecting the preference for edible weeds in anthropogenic habitats. Raw snacks, including seeds of *Onopordum acanthium* and flowers of *Onosma hispida* represent culturally embedded foraging practices, particularly for children and pastoral communities [47, 53] comparable to observations in Kohistan [76] and Upper Dir [75]. Novelty analysis identified 20 entirely new edible species and eight taxa with new consumption methods such as raw *Euphorbia prostrata* and *Vicia hirsuta* [44], highlighting Swat’s dynamic and evolving food knowledge relative to neighboring regions [74–84].

### Ethnoecology and knowledge transmission

Cross-cultural comparisons among Swat’s multi-ethnic communities (Yousafzai Pathans, Kohistanis, Gujjars, Torwalis, and Shepherds) reveal heterogeneous knowledge transmission influenced by linguistic diversity, mobility, and social structures. Informants from tehsils like Bahrain (Kohistani/Gujjar dominant) shared common vernacular names for *Berberis lycium* “Ghwaraskey” and *Chenopodium album* “Sarmay”, while usage variations such as *Mentha royleana* in sauces versus salads reflect adaptation to micro-ecological conditions and inter-ethnic negotiations [18, 46]. Elderly informants (56.25% aged 50–90) retained most ethnobotanical knowledge emphasizing the significance of intergenerational transfer. Gendered knowledge distribution showed men (62.5%) excelled in plant collection and outdoor foraging, whereas women specialized in preparation and household-level knowledge transmission [33, 40, 70], a pattern similarly documented in Kumrat [75], Kohistan [76], Gadoon Valley [79], and Balochistan [82–84]. Linguistic diversity enriched plant nomenclature and ecological understanding, although dominant languages may contribute to erosion of minority knowledge [22].

### Food–Medicine link and public health relevance

Approximately 80% of WEPs were reported to have medicinal properties, highlighting their dual role as nutritional and therapeutic resources. *Berberis lycium* (cooling beverage, digestive aid), *Cordia* spp. (diabetes management), and *Moringa oleifera* (general health promotion) illustrate the food–medicine continuum [19, 62]. Comparable dual-use species are noted in Kumrat [75], Kohistan [76], and Upper Dir [77], though Swat uniquely documents several novel raw consumption practices. The decline in WEP availability due to overharvesting, habitat degradation, and climate pressures threatens dietary diversity and public health, disproportionately affecting children, the elderly and food-insecure households.

### Knowledge erosion and environmental pressure

Erosion of traditional knowledge is evident as younger generations increasingly perceive WEP foraging as outdated or associated with poverty [42, 70]. Factors include globalization, market integration, dietary commodification, climate change, and ongoing ecological degradation [54, 69]. Species such as *Kalanchoe* spp., *Lathyrus aphaca*,* Oxalis corniculata*,* Rumex dentatus*, and *Vicia sativa* have disappeared locally paralleling patterns observed in Lalku Valley [74], Kumrat [75], and Hindu Kush tribal regions [78]. Such losses threaten cultural continuity, local food security, and adaptive capacity to climate variability [8, 55].

### Economic significance and sustainability

Twenty-seven species (15%) were marketed, generating USD 0.09–1.74 per kg and supplementing household incomes [3, 58]. Vegetables like *Dryopteris juxtapostia* and fruits such as *Berberis lycium* provide critical income. Endangered taxa (*Abies pindrow*,* Mentha royleana*,* Zanthoxylum armatum*) and vulnerable species (Thymus linearis, Mentha longifolia, Morus alba) face overexploitation [28, 68]. Economic dependence on WEPs is also evident in Kohistan [76], North Waziristan [77], Gadoon Valley [79], and Balochistan [82–84]. Sustainable harvesting, cultivation, and policy support are essential to balance livelihoods with biodiversity conservation.

### Environmental and public health implications

WEPs provide critical micronutrients in a region experiencing acute malnutrition and rising non-communicable diseases. Climate variability, deforestation (1.4–1.5% annually), biodiversity loss, and river pollution exacerbate ecosystem instability [15, 34, 49, 56]. Sensitive species like *Abies pindrow* and *Mentha royleana* are vulnerable to phenological shifts, while aquatic taxa such as *Nasturtium officinale* face bioaccumulation risks [46, 52]. Similar pressures are reported in Kumrat [75], Kohistan [76], North Waziristan [77], and Balochistan [82, 84]. Without targeted interventions, seasonal staples like young shoots (41.7%) and fruits (30.6%) could become scarce, heightening nutritional vulnerability. Food insecurity projections indicate 15–20% increases by 2030 due to climate and socio-economic stressors [5, 29, 57].

### Integrated recommendations and future directions

**Fig. 4:**
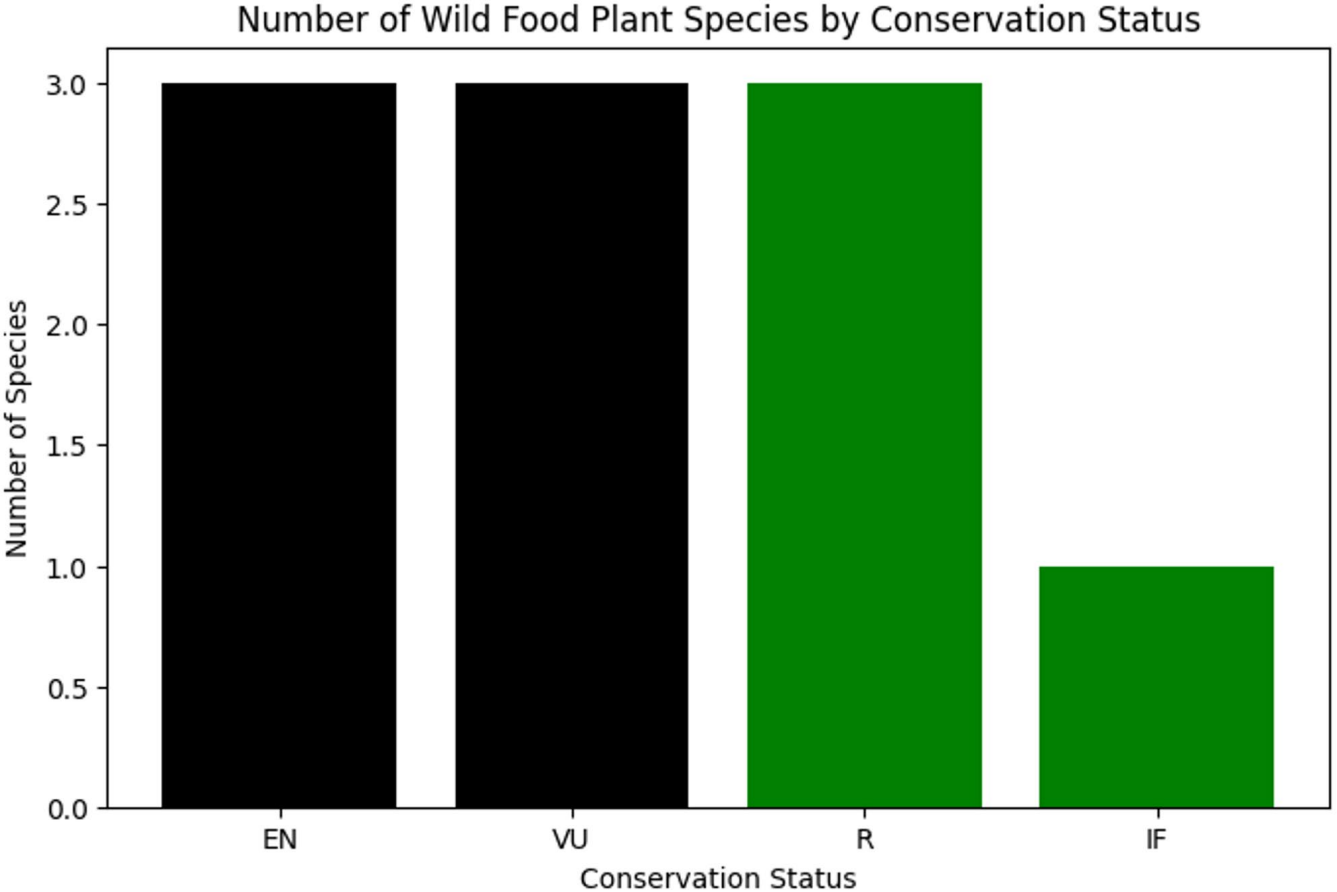
WFPS and their conservation status

Domestication of high-UV species in kitchen gardens, community-led conservation, river pollution monitoring, and nutrition education linking traditional diets to NCD prevention are recommended. Awareness campaigns should highlight ecological, economic, and cultural importance, especially for threatened taxa (EN, VU, R, IF) such as *Abies pindrow*,* Mentha royleana*,* Zanthoxylum armatum*,* Thymus linearis*, and *Morus alba* as shown in Fig. 4. Lessons from Kohistan [76] and Hindu Kush tribal regions [78] illustrate the effectiveness of community-led conservation. Regular monitoring, policy integration, longitudinal studies, and climate-driven modeling are critical to safeguard Swat’s biocultural heritage, ecosystem stability, and public health. Limitations include recall bias and absence of phytochemical validation.

## Conclusion

The present study provides a comprehensive documentation of the diversity, traditional uses, and culinary innovations of wild edible plants in the Swat Valley, Pakistan. A total of 175 plant species from 72 families were recorded, highlighting their significant role in local diets as vegetables, fruits, flavoring agents, teas, sauces, and raw foods. The findings demonstrate that wild plants are not only vital for food security and nutrition, particularly for economically vulnerable households, but also contribute to cultural identity, traditional knowledge, and local livelihoods through market trade. The study revealed notable innovations in the use of certain species, with several globally medicinal or ornamental plants being incorporated into unique culinary practices in Swat, reflecting the region’s rich ethnobotanical knowledge and ecological adaptation.

Moreover, the dependence on wild edible plants underscores the urgent need for sustainable management, conservation, and community engagement to prevent the depletion of valuable species, some of which are already becoming rare. The research highlights the potential consequences of climate variability, habitat degradation, and overharvesting on both biodiversity and public health, emphasizing the importance of integrating traditional knowledge with policy, monitoring, and education programs.

Overall, this research emphasizes that wild edible plants represent an underexplored yet essential component of local food systems, cultural heritage, and ecological sustainability in mountainous regions. These insights provide a foundation for future conservation strategies, nutritional studies, and policy interventions aimed at promoting the sustainable utilization, protection, and resilience of wild plant resources in Swat Valley and similar environments.

## Data Availability

All datasets generated and analyzed during the current study are included in this published article and its supplementary materials. Additional raw data may be provided by the corresponding author upon reasonable request.
